# Dose–Response Relationships Between Exercise Modalities and Triglyceride Reduction in Adults: A Systematic Review and Model-Based Network Meta-Analysis

**DOI:** 10.3390/metabo16090683

**Published:** 2026-09-17

**Authors:** Junru Zeng, Hong Wang, Fengrui Shi

**Affiliations:** School of Wushu, Wuhan Sports University, Wuhan 430079, China; 15153889066@163.com (J.Z.); 18339191039@163.com (F.S.)

**Keywords:** triglycerides, exercise, metabolic equivalent, dose–response, network meta-analysis, randomized controlled trial

## Abstract

**Highlights:**

**What are the main findings?**

- Among 76 randomized trials, AE, CE, and HIIT reduced TG; CE produced the largest average network estimate, whereas the effect of RT was inconclusive.
- TG reductions increased approximately linearly across observed exercise doses, with no supported plateau or interior optimal dose.

**What are the implications of the main findings?**

- Exercise prescriptions should prioritize feasible, modality-specific dose ranges rather than a single universal optimal dose.
- Estimates at the upper dose boundaries, particularly for CE and HIIT, should be interpreted cautiously and confirmed in dose-ranging trials.

**Abstract:**

Background/Objectives: Triglyceride-rich lipoproteins contribute to residual atherosclerotic cardiovascular risk, but the comparative and dose–response effects of exercise modalities on triglycerides (TGs) remain uncertain. We compared aerobic exercise (AE), resistance training (RT), combined exercise (CE), and high-intensity interval training (HIIT) and characterized modality-specific dose–response relationships. Methods: Five databases were searched from inception through January 2026. Randomized trials in adults were synthesized using contrast-based random-effects network meta-analysis (NMA) and model-based network meta-analysis (MBNMA), with dose harmonized as MET·min/week. Results: Seventy-six independent trials included 4483 participants in 208 randomized arms. The 122 active arms generated 122 exercise–control contrasts; one CE contrast with zero reported sampling variance was excluded from both network models, leaving 121 analyzed contrasts in 86 independent comparator blocks. Exercise reduced TG by 0.149 mmol/L, on average (95% CI 0.099–0.199), although the prediction interval crossed the null. CE had the largest average network estimate (MD −0.255 mmol/L), followed by HIIT (−0.225) and AE (−0.130); RT was inconclusive. The modality-specific linear model had the lowest AICc. Per 1000 MET·min/week, slopes were −0.128 mmol/L for AE, −0.307 for CE, −0.081 for RT, and −0.239 for HIIT; the RT slope crossed the null. No supported interior optimum was identified. Conclusions: AE, CE, and HIIT were associated with lower TGs, but certainty was low, and 50 trials were at high risk of bias. CE produced the largest average estimate within the available network, not definitive evidence of universal superiority. The reported lowest and upper observed doses are descriptive boundaries rather than efficacy thresholds or prescription targets.

## 1. Introduction

Triglycerides (TGs) are the principal lipid constituent of chylomicrons and very-low-density lipoproteins. Although circulating TGs are a biomarker rather than the sole atherogenic moiety, converging epidemiological, genetic, and mechanistic evidence implicates TG-rich lipoproteins and their remnants in atherosclerotic cardiovascular disease (ASCVD) [1,2,3]. Remnant particles can penetrate and become retained within the arterial wall, promote inflammation, and contribute cholesterol to developing plaques [1,2]. Hypertriglyceridemia is also closely linked to insulin resistance, ectopic fat accumulation, obesity, type 2 diabetes, and non-alcoholic fatty liver disease, making TGs a clinically relevant target across diverse metabolic phenotypes [3,4].

Lifestyle modification remains a first-line strategy for managing elevated TGs. Exercise may reduce circulating TGs through increased skeletal-muscle fatty-acid oxidation, greater lipoprotein-lipase activity, improved insulin sensitivity, reduced hepatic very-low-density lipoprotein production, and favorable changes in body composition [5,6,7]. Meta-analyses of randomized trials indicate that exercise training produces modest average reductions in TGs, but responses vary substantially among trials and populations [8,9,10,11]. This heterogeneity is biologically plausible because aerobic, resistance, combined, and interval exercise impose different energetic and neuromuscular demands. It may also reflect variation in baseline TGs, adiposity, metabolic disease, medication use, diet, exercise supervision, and adherence.

Previous syntheses have mainly estimated an average effect for a modality or examined isolated training variables in conventional meta-regression [8,9,10,11]. Such approaches are useful but do not fully characterize how TG changes across the doses actually studied for each modality or whether the data support curvature, a plateau, or an interior optimum. Categorizing continuous dose into arbitrary low, moderate, and high groups discards information, while treating every modality–dose combination as an unrelated intervention can fragment the network and reduce precision.

Model-based network meta-analysis integrates the relative-treatment framework of network meta-analysis with biologically plausible dose–response functions [12,13,14]. It preserves randomized comparisons, borrows strength across doses, enables predictions at doses not directly studied, and can formally compare candidate functional forms. Exercise protocols can be placed on a common exposure scale using metabolic equivalent task minutes per week (MET·min/week) while acknowledging that MET assignment introduces measurement uncertainty [15,16].

Accordingly, this systematic review and NMA aimed to (1) compare the effects of AE, RT, CE, and HIIT on TG; (2) estimate overall and modality-specific dose–response relationships; (3) report the lowest observed dose and the range supported by available data, in addition to assessing whether an interior optimum or threshold was supported; and (4) examine whether participant and intervention characteristics modified treatment effects. We hypothesized that exercise would lower TGs relative to the non-exercise control and that the magnitude and shape of the dose–response relationship would differ across modalities.

## 2. Materials and Methods

This review is reported in accordance with PRISMA 2020 and the PRISMA extension for network meta-analyses [17,18]. The protocol was registered in PROSPERO (CRD420261469330). The completed PRISMA-NMA checklist is provided in Table S2.

### 2.1. Eligibility Criteria

The eligibility criteria are summarized in Table 1.

### 2.2. Information Sources and Search Strategy

PubMed, Web of Science Core Collection, Embase, Scopus, and the Cochrane Library were searched independently by two reviewers (J.R.Z. and F.R.S.) from inception through January 2026, without language restrictions. The strategy combined controlled vocabulary and free-text terms for exercise, triglycerides, and randomized trials. Reference lists of eligible reports and relevant systematic reviews were screened. Complete database-specific strategies and the date each search was run are provided in Table S1.

### 2.3. Study Selection Process

After deduplication, two reviewers (J.R.Z. and F.R.S.) independently screened titles/abstracts and full texts. Reasons for full-text exclusion were recorded. Reports arising from the same underlying trial were linked and treated as one independent trial; the most complete report supplied outcome data, while companion reports supplied intervention or participant details when needed. Disagreements were resolved by consensus or by a third reviewer (H.W.). The selection process was documented using a PRISMA 2020 flow diagram.

### 2.4. Data Extraction and Outcome Harmonization

Two reviewers independently extracted study identifiers, country, design, recruitment setting, participant characteristics, eligibility criteria, sample size, age, sex, BMI, baseline health status, lipid-modifying medication, diet instructions, exercise supervision and adherence, intervention duration, frequency, session duration, intensity, modality, and TG values. Each randomized arm occupied one row in the analysis dataset. Multiple eligible exercise arms were retained, and shared comparators were handled using multi-arm covariance structures rather than double-counting participants.

TGs were analyzed in mmol/L. Values reported in mg/dL were divided by 88.57. Standard errors were converted to standard deviations (SDs) using SD = SE × √*n*. For a 95% confidence interval around a mean, the SD was derived from the interval width and the appropriate t distribution. When only medians and dispersion measures were available, means and SDs were estimated using validated methods and examined in sensitivity analysis [19]. Values digitized from figures were independently extracted twice and flagged. Adjusted post-intervention means were not pooled with unadjusted change scores in the primary analysis unless their estimands were judged compatible.

The preferred outcome was the between-group difference in change from baseline. If the change-score SD was unavailable, it was calculated as SD_change_ = √(SD_pre_^2^ + SD_post_^2^ − 2*r*·SD_pre_·SD_post_), using a prespecified within-person correlation estimated from trials reporting complete data; alternative correlations (0.25, 0.50, and 0.75) were examined [20]. For trials reporting only post-intervention values, a post-value analysis was used in sensitivity analysis because randomization should balance baseline values on average.

### 2.5. Exercise Classification and Dose Assignment

Exercise arms were classified according to their prescribed structure and the American College of Sports Medicine framework [16]. AE comprised continuous endurance activity, RT comprised planned muscle contractions against resistance without a planned aerobic component, CE included both planned aerobic and resistance components, and HIIT comprised repeated vigorous or high-intensity work intervals separated by recovery. Actual prescribed components governed classification rather than a broad label or presumed dominant stimulus. Tai chi, yoga, and other mind–body interventions were not included in the review. Control arms were coded as CG and assigned a dose of 0 MET·min/week.

Weekly dose was calculated as MET value × session duration (min) × frequency (sessions/week). For sessions containing components with different MET values, component doses were summed. Activity-specific MET values were assigned from the 2024 Adult Compendium of Physical Activities [15]. When only intensity was reported, midpoint MET values from prespecified absolute-intensity categories were applied and documented. For older adults or clinical populations, assigned MET values represented standardized prescribed external exposure rather than individualized oxygen cost or achieved physiological load. Uncertain or imputed doses were flagged and examined using lower/upper plausible-dose sensitivity analyses.

### 2.6. Risk-of-Bias Assessment

Two reviewers (J.R.Z. and F.R.S.) independently assessed the TG result from each trial using the revised Cochrane Risk of Bias tool (RoB 2) across five domains: randomization process, deviations from intended interventions, missing outcome data, outcome measurement, and selection of the reported result [21]. Domain-level judgments and the overall judgment (low risk, some concerns, or high risk) were supported by trial-specific justifications. Because participant and personnel blinding is generally infeasible in exercise trials, judgments focused on whether deviations, co-interventions, attrition, and analysis choices plausibly biased TG estimates; laboratory TG measurement, itself, was objective.

### 2.7. Effect Measures and Conventional Meta-Analysis

The effect measure was the mean difference (MD) in TG change (mmol/L), coded so that negative values favored exercise. For each sufficiently represented modality, pairwise random-effects meta-analysis summarized exercise versus control. Statistical heterogeneity was described using τ^2^, I^2^, and 95% prediction intervals. Small-study effects were examined using contour-enhanced funnel plots and Egger-type regression when at least 10 independent comparisons contributed to an analysis [22,23].

### 2.8. Network Meta-Analysis

A contrast-based random-effects network meta-analysis was fitted by generalized least squares. For each active-versus-control contrast, the sampling variance was Var(Δ_active_)/*n*_active_ + Var(Δ_control_)/*n*_control_. Covariance between two contrasts sharing the same comparator equaled the comparator change-score variance divided by its sample size. Multi-arm random effects used τ^2^ on the diagonal and τ^2^/2 off diagonal. τ^2^ was estimated by restricted maximum likelihood for the principal network models. Transitivity was evaluated by comparing baseline TG, age, sex, BMI, clinical status, duration, co-interventions, and risk of bias across comparisons. Relative effects were reported with 95% confidence and prediction intervals; ranking probabilities were secondary [24].

### 2.9. Dose–Response Model-Based Network Meta-Analysis

Dose–response MBNMA was fitted using contrast-based GLS while preserving randomization and shared-comparator covariance [12,13,14]. Dose was expressed per 1000 MET·min/week, and all functions passed through zero at the control dose. Four prespecified candidates were fitted: common linear, common quadratic, modality-specific linear, and modality-specific quadratic. Models were estimated by maximum likelihood and compared using AIC and AICc. The selected function was required to improve fit without relying on unsupported curvature at dose boundaries.

Model fit was evaluated using maximized log likelihood, AIC, AICc, residual heterogeneity, and visual agreement between fitted curves and observed contrasts. For a supported nonlinear model, an interior optimum would be defined as the dose with the greatest predicted TG reduction within the observed range. For a selected monotonic model, no interior optimum was claimed; instead, slopes, observed ranges, the lowest studied dose on a statistically supported slope, and effects at the upper observed boundary were reported. Extrapolation beyond observed modality-specific ranges was prohibited. Parameter uncertainty was propagated using 20,000 multivariate-normal draws from the estimated coefficient covariance matrix.

### 2.10. Meta-Regression, Subgroup, and Sensitivity Analyses

When data were sufficient, meta-regression examined baseline TGs, age, BMI, proportion of women, metabolic disease status, intervention duration, supervision, and publication year. Subgroup analyses distinguished metabolically healthy participants from those with obesity, diabetes, metabolic syndrome, dyslipidemia, and fatty liver disease. Sensitivity analyses excluded high-risk-of-bias trials, imputed change-score SDs, estimated MET values, figure-derived outcomes, co-intervention trials, interventions shorter than 8 weeks, and trials with lipid-lowering medication imbalance. Leave-one-study-out analyses assessed influential observations, and restriction to doses no greater than 2000 MET·min/week assessed sparse high-dose leverage.

### 2.11. Certainty of Evidence and Software

Confidence in each decision-relevant network estimate was evaluated using the CINeMA domains as a structured framework: within-study bias, reporting bias, indirectness, imprecision, heterogeneity, and incoherence [25]. Formal automated CINeMA computation was not possible for all predominantly star-network contrasts, so domain judgments were reported transparently and conservatively. Analyses were conducted in Python 3.12 (Python Software Foundation, Wilmington, DE, USA) using NumPy 2.1, SciPy 1.14, pandas 2.2, and Matplotlib 3.9.

## 3. Results

### 3.1. Study Selection

**Figure 1:**
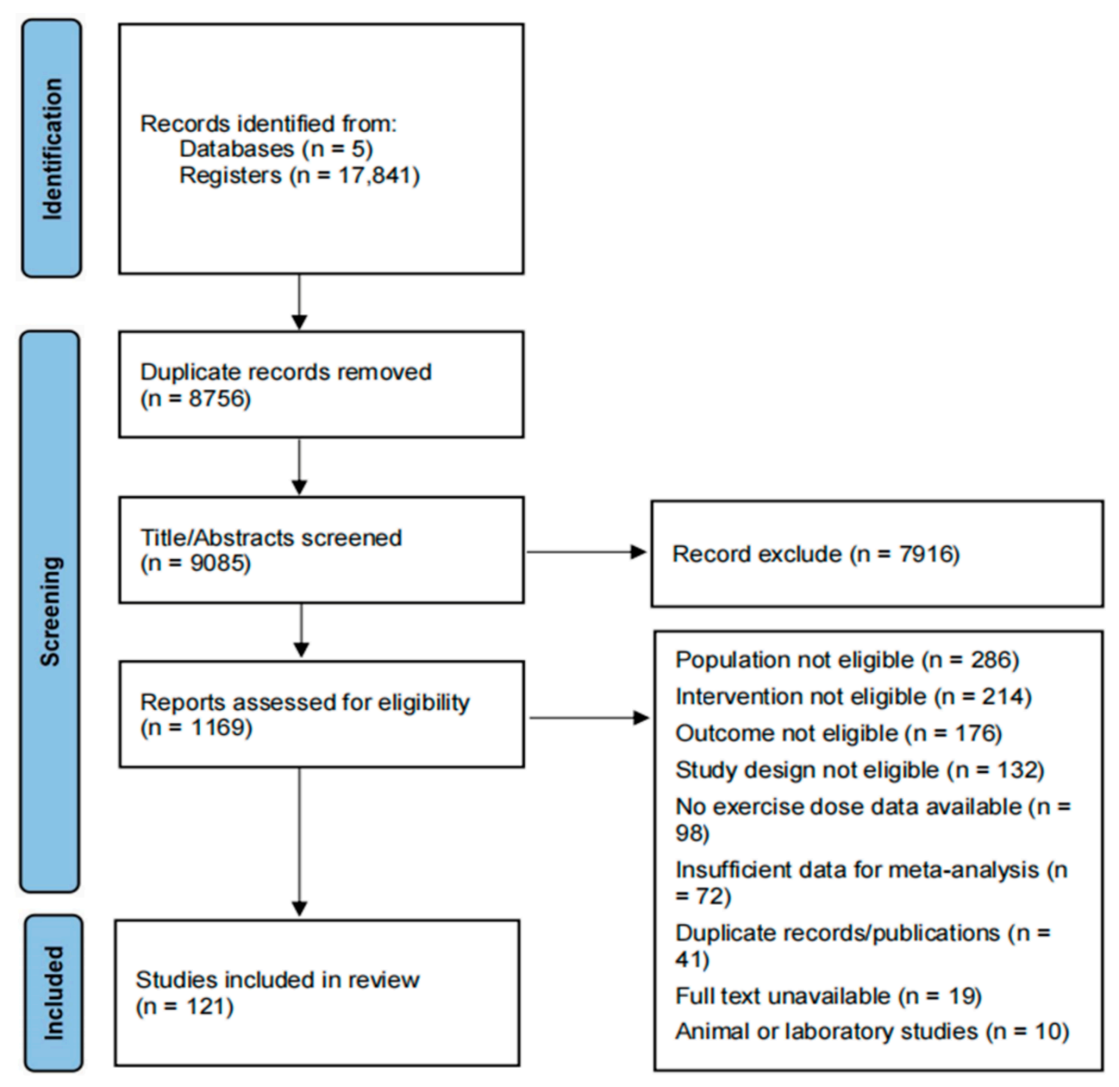
PRISMA flow diagram of study identification, screening, eligibility assessment, and inclusion.

The search yielded 121 eligible reports, which were linked to 76 independent randomized trials before analysis (Figure 1). These trials comprised 208 randomized arms and 4483 participants: 122 active exercise arms and 86 control arms. The 122 active arms generated 122 active–control contrasts. One CE contrast from LeMura et al. [26] had a reported sampling variance of zero and was excluded from both the NMA and MBNMA, leaving 121 analyzed contrasts in 86 independent comparator blocks. Table S10 provides the evidence-unit accounting and reconciliation.

### 3.2. Study and Participant Characteristics

The 76 trials were published from 1979 to 2025. The descriptive dataset included 74 AE arms (1777 participants), 20 CE arms (473 participants), 20 RT arms (329 participants), 8 HIIT arms (121 participants), and 86 control arms (1783 participants). One CE arm was excluded only from quantitative models because its derived contrast had zero sampling variance; thus, 19 CE contrasts entered the NMA and MBNMA. The median intervention duration was 12 weeks for AE, 16 weeks for CE, 15 weeks for RT, and 10 weeks for HIIT. The registered protocol prespecified a minimum intervention duration of 2 weeks. The shortest included interventions lasted 3 weeks: Smith-Ryan et al. [27] contributed two 3-week HIIT contrasts; no eligible 2-week trial was identified. Taniguchi et al. [28] contributed one 5-week AE contrast. The ≥8-week sensitivity analysis excluded these three contrasts and retained 74 trials and 118 contrasts. Observed doses ranged from 90 to 2835 MET·min/week for AE, 53 to 1800 for CE, 315 to 1080 for RT, and 660 to 1161 for HIIT. Detailed trial characteristics are provided in Table S3, and summary arm-level characteristics are shown in Table 2.

### 3.3. Risk of Bias

No trial received an overall low-risk judgment. Twenty-six trials (34.2%) raised some concerns, and 50 (65.8%) were judged as high risk. The overall distribution was driven mainly by limitations in randomization reporting and missing outcome data; outcome measurement was generally low risk because TG was assessed objectively in blood samples. Detailed domain judgments appear in Table S4 and Figure S2.

### 3.4. Pairwise and Network Meta-Analysis

**Figure 2:**
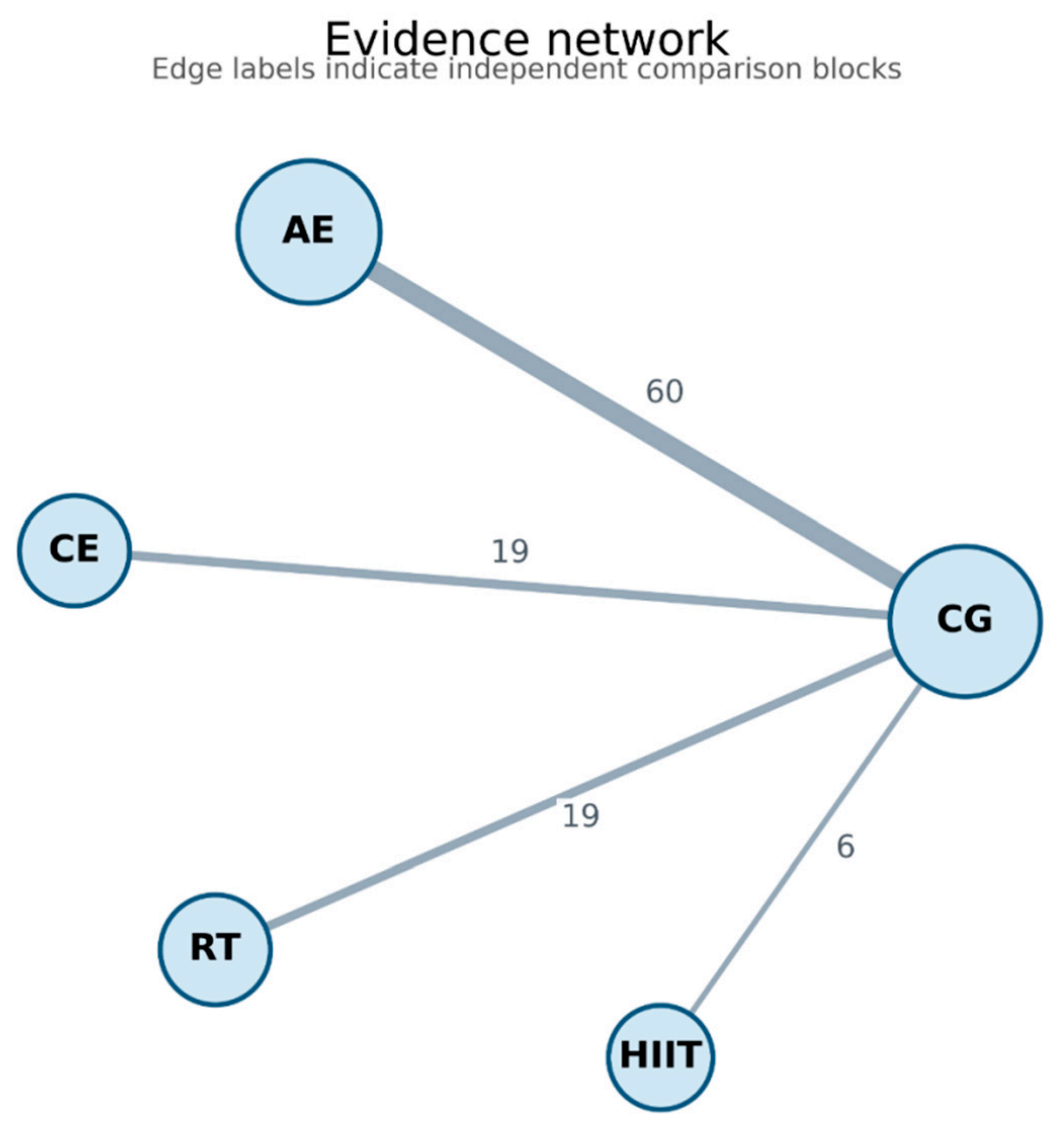
Evidence network. Node sizes reflect the number of randomized arms. Edge labels count unique modality-by-comparator-block links after multiple same-modality arms sharing one comparator were counted once: AE, 60; CE, 19; RT, 19; and HIIT, 6. These 104 links are not the same unit as the 121 analyzed arm-level contrasts.

The evidence network is shown in Figure 2. Full pairwise random-effects results are provided in Table S6.

**Figure 3:**
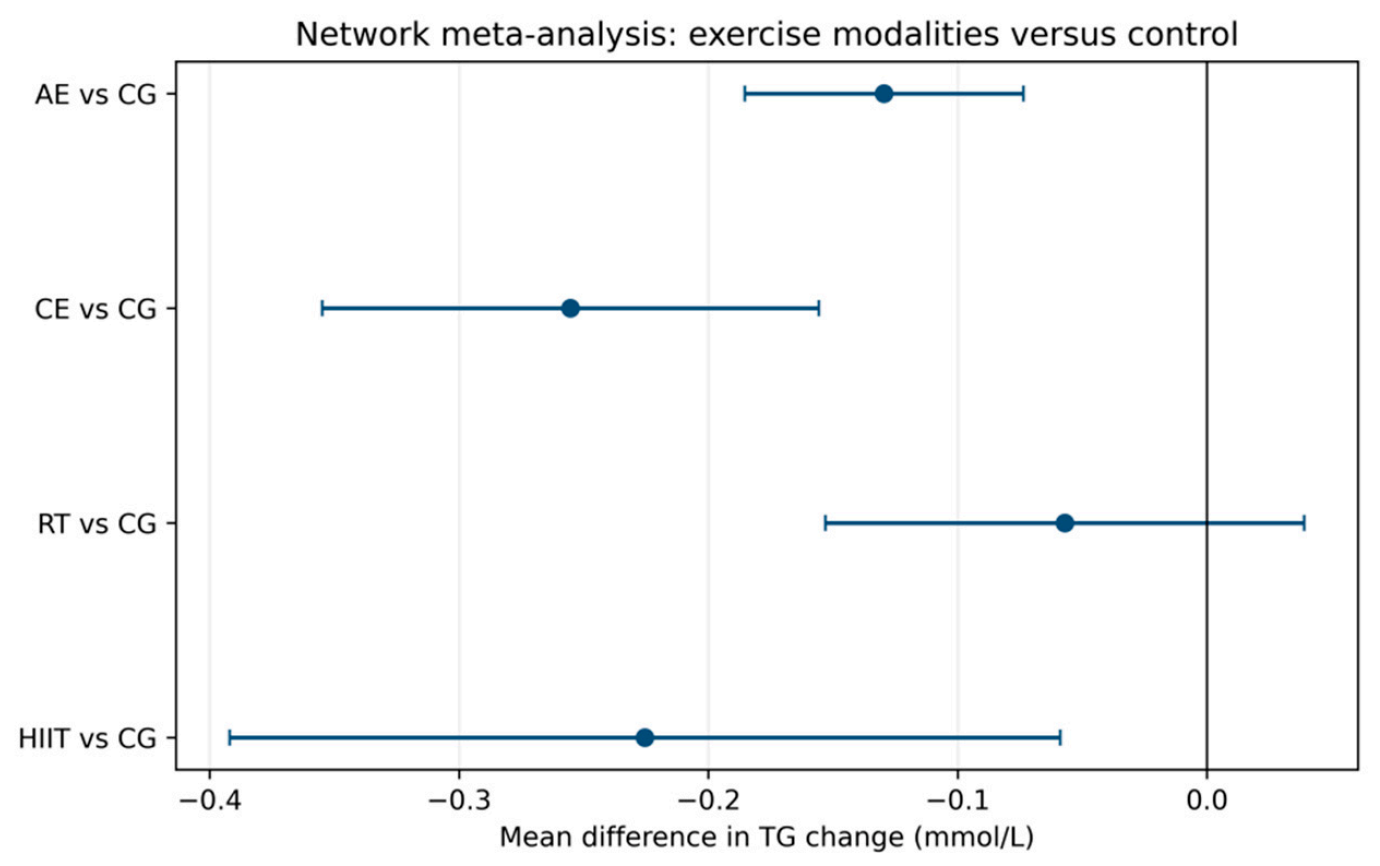
Network meta-analysis estimates for triglyceride change by exercise modality versus control. Negative values favor exercise; points are mean differences, and horizontal lines are 95% confidence intervals. Prediction intervals are reported in Table 3.

The random-effects synthesis of all exercise modalities yielded an MD of −0.149 mmol/L (95% CI −0.199 to −0.099; τ = 0.106 mmol/L; I^2^ = 7.8%). The 95% prediction interval (−0.362 to 0.064) crossed zero, indicating that a future trial in a different clinical context may observe little or no TG reduction despite a favorable mean effect. In the modality network, CE had the largest average estimate (MD −0.255, 95% CI −0.355 to −0.156), followed by HIIT (−0.225, −0.392 to −0.059) and AE (−0.130, −0.185 to −0.074); RT was inconclusive (−0.057, −0.153 to 0.039). Only the CE prediction interval excluded the null (−0.455 to −0.055). Active–active estimates favored CE over AE (CE minus AE −0.126 mmol/L, 95% CI −0.234 to −0.018) and CE over RT (−0.198, −0.331 to −0.065); other active–active estimates were imprecise (Table S7). These are network estimates with limited direct head-to-head evidence and low certainty, so they do not establish universal modality superiority. Ranking probabilities were treated as secondary. Modality-specific estimates versus control are displayed in Figure 3 and summarized in Table 3. Ranking probabilities are shown in Figure S6.

### 3.5. Dose–Response MBNMA

**Figure 4:**
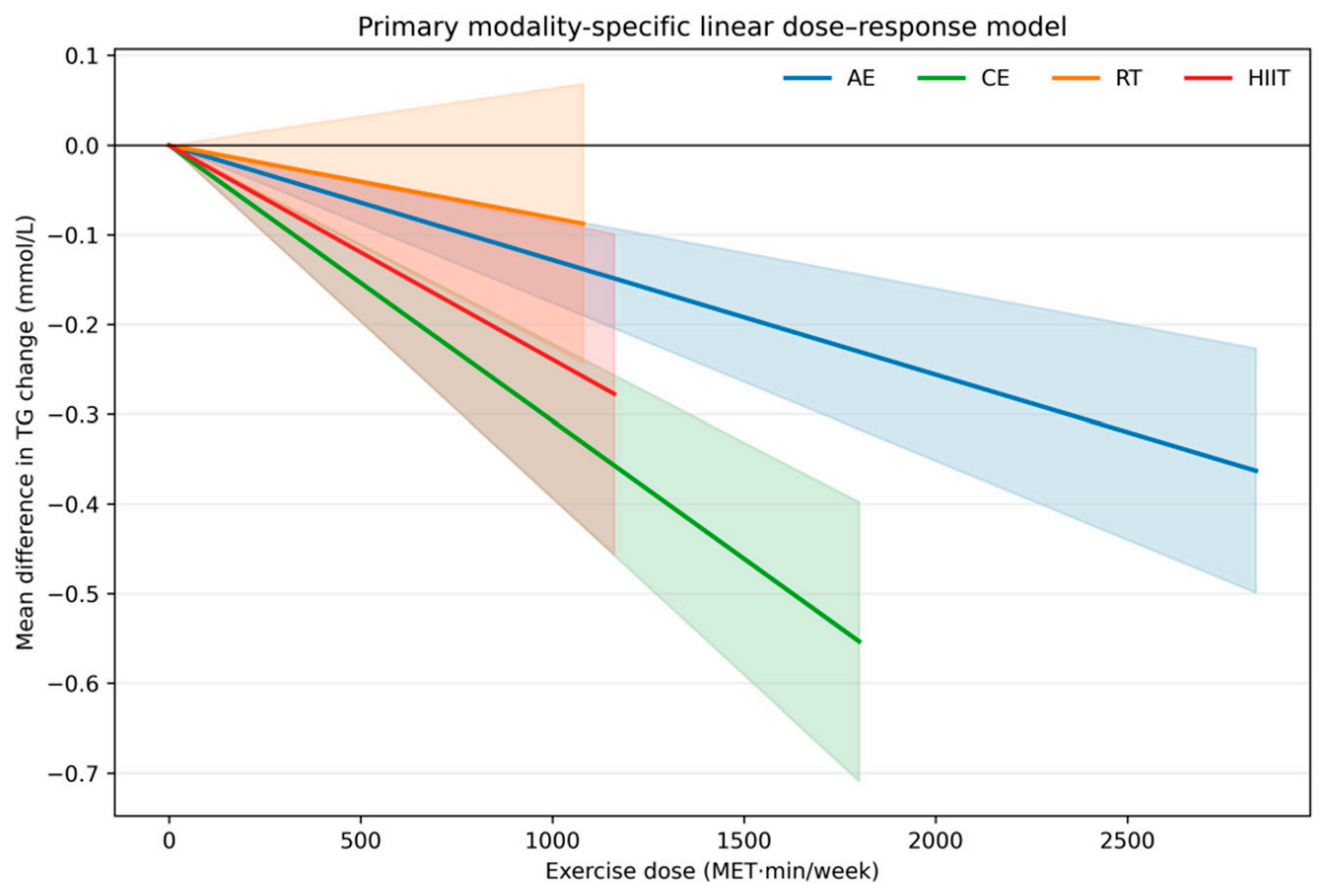
Primary modality-specific linear dose–response relationships. Shaded bands are 95% confidence intervals for the fitted conditional mean; negative values indicate larger TG reductions. Curves are displayed only over each modality’s observed dose range, and sparse boundary regions should be interpreted cautiously.

Four candidate functions were compared. The modality-specific linear model provided the lowest AICc (−0.03), ahead of the modality-specific quadratic (5.47), common linear (10.29), and common quadratic (10.33) models. Their respective parameter counts were 4, 8, 1, and 2; maximized log likelihoods were 5.278, 7.077, −3.095, and −2.063; and residual heterogeneity estimates (τ) were 0.065, 0.052, 0.102, and 0.097 mmol/L (Table S5). The selected model estimated TG changes per 1000 MET·min/week of −0.128 mmol/L (95% CI −0.176 to −0.080) for AE, −0.307 (−0.394 to −0.221) for CE, −0.081 (−0.225 to 0.063) for RT, and −0.239 (−0.393 to −0.085) for HIIT. No interior optimum or plateau was supported. Conditional mean predictions at the upper observed boundaries were recorded as follows: AE, −0.363 mmol/L (95% CI −0.499 to −0.227) at 2835 MET·min/week; CE, −0.553 (−0.709 to −0.398) at 1800; RT, −0.087 (−0.243 to 0.068) at 1080; and HIIT, −0.278 (−0.456 to −0.099) at 1161. These confidence intervals describe uncertainty in the fitted mean curve, not between-study prediction intervals. The lowest observed doses on statistically supported slopes were 90 MET·min/week for AE, 53 for CE, and 660 for HIIT; they are observations at data boundaries, not biological thresholds or minimum prescriptions. The fitted slopes and upper-bound conditional mean effects are summarized in Table 4, and the fitted dose–response curves are shown in Figure 4.

### 3.6. Meta-Regression and Sensitivity Analyses

Sensitivity analyses confirmed the overall mean effect after excluding high-risk trials (MD −0.167, 95% CI −0.234 to −0.100), restricting to interventions lasting at least 8 weeks (−0.149, −0.200 to −0.099), and excluding doses above 2000 MET·min/week (−0.149, −0.200 to −0.099). The ≥8-week analysis retained 74 trials and 118 contrasts, and the ≤2000 MET·min/week analysis retained 75 trials and 119 contrasts. Heterogeneity fell after exclusion of high-risk trials (τ 0.037 versus 0.106 mmol/L), suggesting that methodological limitations contributed to between-trial dispersion. Leave-one-study-out estimates did not reverse the direction of the pooled effect, and the high-dose restriction showed that the overall estimate was not driven by the sparsely populated region above 2000 MET·min/week. Modality-specific boundary predictions, especially for CE and HIIT, nevertheless remain vulnerable to sparse-dose leverage. The exploratory comparison-adjusted Egger regression was significant (intercept −0.269; *p* = 0.018), consistent with small-study effects or selective reporting; because contrasts within multi-arm trials are correlated, this result was treated as a signal rather than definitive evidence of publication bias. Complete sensitivity analyses are provided in Table S8. The observed dose distribution, comparison-adjusted funnel plot, and dose–response sensitivity analyses are shown in Figures S1 and S3–S5, respectively.

### 3.7. Confidence in the Evidence

Confidence was low for AE and CE because favorable estimates were tempered by high within-study risk of bias, possible small-study effects, clinical indirectness across heterogeneous populations, and—in CE—substantial pairwise heterogeneity. Confidence was low for HIIT because only six trials and eight analyzed contrasts informed the estimate and its prediction interval crossed the null. Confidence was very low for RT because the mean effect and dose slope were imprecise and risk of bias was substantial. Accordingly, CE should be described as having the largest average network estimate, not as definitively superior, and all modality-specific clinical translations should retain the associated certainty rating and prediction uncertainty. Confidence assessments for modality-versus-control comparisons are provided in Table S9.

## 4. Discussion

### 4.1. Principal Findings

This systematic review of 76 randomized trials indicates that structured exercise lowers TGs by approximately 0.15 mmol/L, on average. CE had the largest average network estimate, but the evidence was low-certainty, and limited direct head-to-head data preclude a definitive claim of superiority. HIIT and AE also had favorable mean estimates; RT remained inconclusive. The dose analysis supported modality-specific linear trends over observed ranges but no interior optimum or biological threshold. The lowest and highest observed-dose predictions are therefore boundary descriptions that should inform future trials, not minimum or optimal prescriptions.

### 4.2. Interpretation in Relation to Previous Evidence

Baseline TGs are likely one of the most important unmeasured determinants of absolute response. Participants entering a trial with elevated TGs have greater biological room for reduction and may show larger mmol/L changes than normotriglyceridemic participants, even when relative changes are similar. Pooling an absolute MD is clinically interpretable and avoids the instability of percentage change near low baselines, but it can amplify heterogeneity when baseline risk varies. Aggregate reporting prevented a consistent interaction analysis across all trials. Future updates should prespecify individual-level or arm-level baseline-TG strata and, where possible, jointly model baseline concentration and change rather than relying on study-level meta-regression. In the HERITAGE cohort, men with elevated baseline TG experienced an approximately 15% TG reduction after endurance training, whereas lipid responses differed in those without the high-TG phenotype [29]. Familial aggregation analyses from the same research program attributed a meaningful proportion of lipid-response variance to heritable factors [30], and genomic/transcriptomic work subsequently identified molecular predictors of TG response [31]. These data reinforce that a common exercise dose can yield materially different biochemical responses.

Earlier meta-analyses consistently suggest that exercise training modestly lowers TGs, although estimates vary with population, modality, and analytical choices [8,9,10,11]. The present analysis adds comparative and dose–response resolution while retaining randomization and shared-comparator covariance. The apparent between-modality differences, including CE’s larger average estimate, should be interpreted alongside confidence intervals, prediction intervals, low certainty, and limited direct evidence rather than as a universal hierarchy.

The magnitude of the overall effect is clinically plausible. A reduction of 0.149 mmol/L corresponds to approximately 13.2 mg/dL, using 88.57 mg/dL per mmol/L. This is close to the average TG reduction reported in recent broad exercise meta-analyses, but our prediction interval shows why the mean alone is insufficient. The interval included a small unfavorable effect, implying that context—baseline TGs, achieved dose, diet, medication, and metabolic phenotype—can dominate the average response in a new setting. Prediction intervals should therefore accompany confidence intervals whenever the result is translated into practice.

The comparison with the most recent broad meta-analysis is informative. Smart et al. pooled 148 randomized trials and reported a TG reduction of 8.01 mg/dL (approximately 0.090 mmol/L), whereas the present overall estimate was 0.149 mmol/L [8]. Both analyses therefore support a modest average benefit, and both identify the combination of aerobic and resistance training as the most favorable modality. The somewhat larger estimate here should not be interpreted as superiority of the present method: our eligibility criteria, participant case mix, handling of multi-arm covariance, outcome reconstruction, and explicit inclusion of HIIT differ from those of Smart et al. [8]. Moreover, our 95% prediction interval crossed the null, paralleling their observation that TG did not improve in every trial. The agreement is consequently stronger for the direction and modest scale of benefit than for a single universal effect size.

Recent population-specific syntheses further explain the range of reported effects. Aerobic exercise has been associated with improved triglycerides and other metabolic-syndrome components in older adults with type 2 diabetes [9] and with favorable cardiometabolic changes in postmenopausal women [10]. A 2025 meta-analysis likewise found that aerobic exercise reduced triglycerides in adults with overweight or obesity [32]. These recent reviews support baseline metabolic status, sex, age, and population composition as effect modifiers rather than implying that one pooled estimate is universally applicable. An updated systematic review also supports favorable aerobic-exercise effects on the lipid profile [33].

The modality findings also show both continuity and an advance over prior evidence. Smart et al. found significant triglyceride reductions after aerobic and combined training but not resistance training [8], closely matching our AE, CE, and RT pattern. A systematic review in adults with metabolic syndrome also compared aerobic, resistance, and combined training [34]. A network meta-analysis in metabolic syndrome also ranked combined exercise as the most effective option for triglyceride control [35], and a meta-analysis in healthy women similarly favored combined training [36]. Meta-analyses focused on resistance training have also reported population-dependent lipid effects [37,38]. More recent syntheses in type 2 diabetes found that concurrent strength and endurance training improved lipid profiles [39,40], while a network meta-analysis identified modality-specific differences in lipid responses [41]. These findings support the direction of our CE estimate but do not establish that the same ordering applies to every clinical population.

Evidence for HIIT is also context-dependent. A 2023 general-population systematic review and meta-analysis found that HIIT improved several cardiometabolic outcomes, although effects varied across outcomes and study characteristics [42]. The present network estimate favored HIIT for triglycerides, but differences in intervention duration, interval structure, comparator choice, baseline triglycerides, and the number of triglyceride-reporting trials may influence the estimate. Accordingly, the favorable HIIT result should be interpreted as evidence of potential efficacy in the populations represented by the network, not proof that HIIT is uniformly superior to continuous aerobic exercise.

Our network synthesis increases comparative resolution by retaining shared comparators and estimating active–active contrasts, but it does not turn rankings into proof of superiority. CE ranked first, yet uncertainty, limited direct head-to-head evidence, and the predominance of high-risk trials require caution. The dose analysis adds a distinct contribution because previous meta-regressions generally examined isolated FITT components. Expressing exposure on a common MET·min/week scale revealed statistically supported modality-specific slopes but no supported interior optimum. Evidence that postprandial TG lowering depends partly on exercise energy expenditure provides physiological support for treating total dose as meaningful [43]; however, converting heterogeneous prescriptions to MET·min/week cannot capture interval structure, internal load, or adherence. Thus, the evidence supports a graded association within observed ranges, not a single threshold or maximally effective dose applicable to all adults.

Biologically, TG reduction after exercise may reflect several related but non-identical processes. Human tracer evidence shows that moderate exercise can suppress hepatic VLDL-TG secretion during exercise and recovery, although VLDL-TG oxidation, itself, may not increase [44]. Exercise training can also increase lipoprotein-lipase activity and modify apolipoprotein and lipoprotein metabolism, facilitating TG-rich particle processing [45]. These mechanisms are compatible with improved insulin sensitivity and greater skeletal-muscle fatty-acid use, but the relative contribution of secretion, clearance, and oxidation probably varies by nutritional state, exercise timing, intensity, and baseline metabolic phenotype.

**Figure 5:**
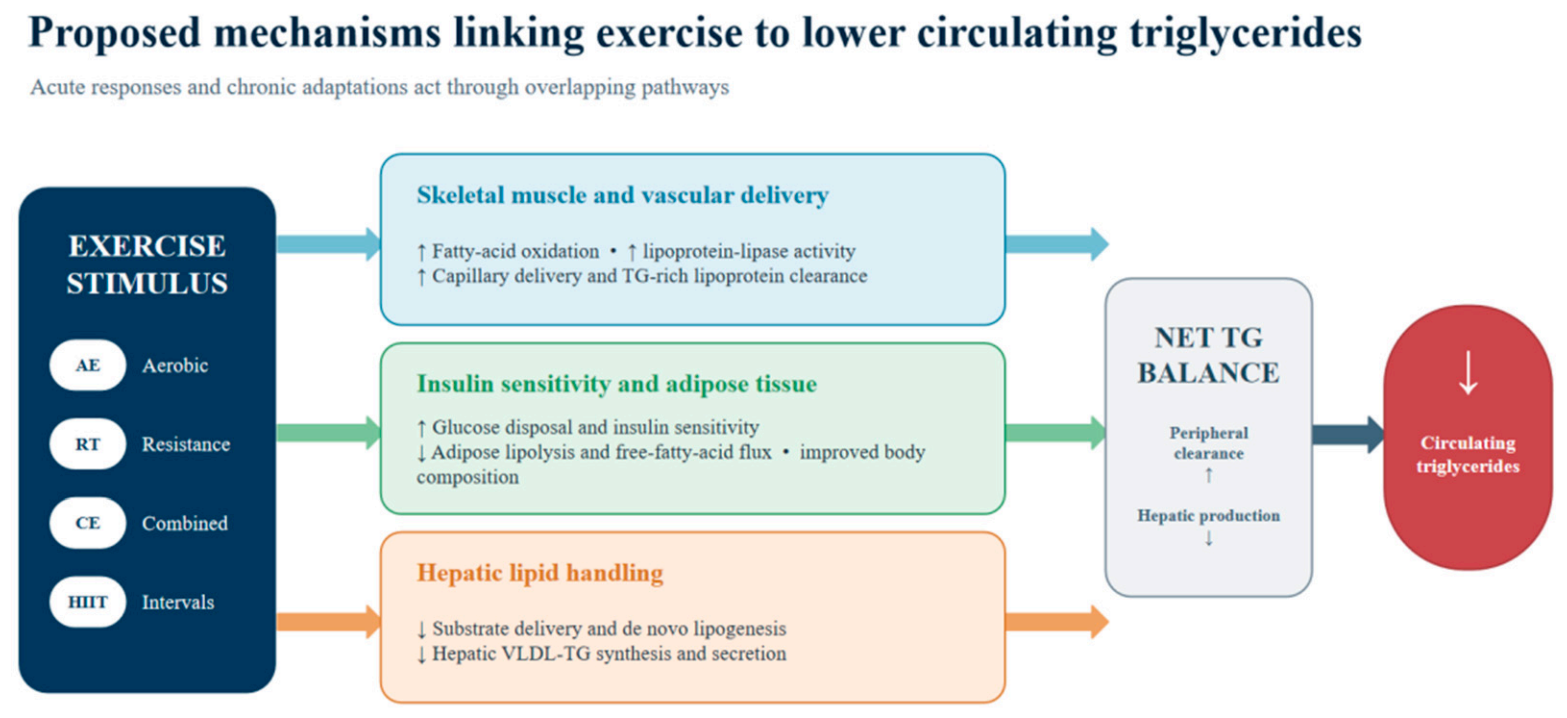
Proposed pathways through which aerobic, resistance, combined, and high-intensity interval exercise may reduce circulating triglycerides. Exercise may increase skeletal-muscle fatty-acid oxidation and lipoprotein-lipase-mediated clearance, improve insulin sensitivity and glucose disposal, alter body composition, and reduce substrate delivery and hepatic VLDL-TG secretion. Arrows indicate the direction of the proposed pathway from exercise stimuli through intermediate metabolic processes to lower circulating triglycerides; upward and downward arrows within boxes indicate increases and decreases, respectively.

The apparently larger CE effect may reflect complementary pathways rather than simple addition of two exercise labels. Aerobic work increases acute and cumulative fatty-acid oxidation and stimulates post-exercise TG clearance; resistance work expands or preserves metabolically active muscle and improves glucose disposal. When combined, these adaptations can reduce substrate delivery to hepatic VLDL production while increasing peripheral clearance, yet CE trials have also delivered comparatively high median exposure and often included longer interventions. Although MBNMA adjusts for dose, incomplete reporting of achieved intensity and adherence leaves residual confounding between “modality” and implementation quality. This interpretation is consistent with comparative syntheses in metabolic syndrome and healthy women, with combined training identified as favorable for TG in both [35,36]. A broader physiological review likewise concluded that aerobic, resistance, and combined exercise can produce different lipid responses because their energetic and muscular stimuli are not interchangeable [46]. These proposed pathways are summarized in Figure 5.

### 4.3. Clinical and Public Health Implications

From a public-health perspective, the finding that benefit is detectable across a broad dose range may be more useful than a single optimum. Population programs must accommodate large differences in baseline fitness, access, culture, disability, and time. A stepped prescription can begin with a feasible dose and progress while monitoring adherence and TG response. Repeated lipid measurement after an adequate training period can identify responders and prompt evaluation of diet, alcohol intake, glycemic control, medication, or genetic dyslipidemia among non-responders. The present curves support progression within studied ranges, but they do not justify making high-volume exercise a prerequisite for care or withholding pharmacological treatment when clinically indicated. The American Heart Association likewise recommends physical activity as a first-line lifestyle treatment for mildly to moderately elevated cardiovascular risk factors and emphasizes that individual lipid responses vary [47].

Exercise modality should be selected in a multi-outcome framework. TGs are important, but a prescription also affects cardiorespiratory fitness, blood pressure, glycemic control, lean mass, strength, mobility, symptoms, and quality of life. CE may be attractive because it addresses several domains, even if the TG-specific advantage is partly dose-related. AE has the largest and most diverse evidence base and may be easiest to scale. RT remains essential for sarcopenia and function despite an inconclusive isolated TG effect. HIIT is time-efficient but requires screening, progression, and supervision in susceptible groups. Shared decision-making should therefore combine TG goals with safety, comorbidity, preference, access, and likelihood of sustained participation. Aerobic training also improves apolipoproteins, lipoprotein subfractions, and atherogenic lipid ratios that are not captured by fasting TG alone [48].

The practical meaning of 1000 MET·min/week depends on activity choice. It corresponds approximately to 222 min of 4.5-MET moderate activity, 154 min at 6.5 MET, or 125 min at 8 MET. These translations are illustrative rather than interchangeable prescriptions: resistance and interval sessions include recovery periods, and their average session MET may differ from peak protocol intensity. Moreover, standardized estimated prescribed exposure is not the same as the dose an individual actually achieves or the internal physiological load experienced. This distinction is especially important in older adults and people with metabolic disease, as well as for RT and HIIT protocols. Clinicians should reconstruct exposure from actual session components, adherence, and tolerance and progress volume gradually.

Clinical translation should prioritize supported ranges rather than a single point estimate. A candidate prescription should lie in a region represented by multiple trials, produce a meaningful predicted TG change, retain acceptable uncertainty, and be feasible for the target population. The lowest observed dose on a supported slope may be described as an entry point for future evaluation, but it is not an efficacy threshold and should not substitute for guideline-recommended activity and its broader health benefits [49]. Even if a TG response eventually plateaus, additional exercise may still improve blood pressure, glycemic control, fitness, mental health, and body composition.

The linear finding needs disciplined interpretation. A straight line was statistically preferred over the tested quadratic alternatives, but this does not prove that benefit can increase indefinitely. It means only that no curvature was reliably detected within the available and unevenly populated dose ranges. Physiological responses must eventually plateau, and very high exercise loads may reduce adherence or increase musculoskeletal and cardiovascular risk. Because the AE and CE greatest-reduction estimates occurred at their maximum observed doses, those values are boundary estimates. Calling them “optimal doses” would convert absence of evidence for a plateau into evidence for unlimited escalation. Classic quantitative evidence suggested that lipid changes can occur at relatively low training volumes but that some outcomes may require a threshold exposure [50]. In a randomized dose study, a higher weekly exercise amount produced broader lipoprotein improvements than lower amounts, whereas intensity was not the principal determinant [51]. A separate meta-analysis estimated an approximate weekly energy-expenditure threshold for increasing HDL-C, illustrating that thresholds may differ across lipid outcomes [52].

The standardized MET·min/week metric facilitates comparison, but it should not obscure individualization. Relative intensity, medication use, baseline fitness, comorbidity, injury risk, and patient preference remain central to prescription. HIIT should not be recommended solely because of a favorable rank if its evidence base is sparse, prediction intervals are wide, or trials enrolled highly selected participants. Similarly, RT may be clinically valuable even when its mean triglyceride effect is smaller because it addresses strength, function, adherence, and other outcomes not captured in this review. Diet and weight change are particularly important co-interventions: a recent meta-analysis in people with obesity and type 2 diabetes found that combining aerobic exercise with dietary intervention improved multiple cardiometabolic outcomes [53].

Interpretation of the lowest observed dose requires caution. In a through-origin linear model, once a slope excludes zero, every positive dose has a model-predicted benefit, and statistical significance is driven by slope precision rather than a genuine efficacy threshold. We therefore report only the lowest studied dose lying on a supported slope: 90 MET·min/week for AE, 53 for CE, and 660 for HIIT. These boundary observations—especially the lowest AE and CE doses—are sparse and should not be promoted as therapeutic minima. The STRRIDE trial further showed that moderate exercise could sustain VLDL-TG lowering after training cessation, while inactivity worsened lipoprotein profiles [54]; this temporal persistence cannot be represented by a model based only on prescribed weekly dose.

### 4.4. Strengths and Limitations

Our effect measure also assumes that the reported pre–post changes and their variances target comparable estimands. Some trials reported complete-case values, others reported intention-to-treat estimates, and several required reconstruction of change-score variance. Correlations between baseline and follow-up TG were rarely available, so derived SDs may be conservative or imprecise. The sensitivity analysis excluding high-risk studies partially addresses this issue but cannot reproduce unavailable participant-level covariance. A future individual-participant-data consortium could harmonize fasting status, medication, baseline TG, and adherence; model repeated measurements directly; and distinguish changes mediated by weight loss from exercise effects independent of weight change. In HERITAGE, fasting TG reduction was most evident 24 h after the final exercise bout and was no longer clearly present at 72 h [55], demonstrating that blood-draw timing relative to the last session can alter the estimand.

An important negative finding is that model flexibility should be earned by data. The modality-specific quadratic model improved raw log likelihood, but its additional parameters were penalized by AICc, and it did not outperform the simpler modality-specific linear model. Full parameter counts, log likelihoods, AIC, AICc, and residual heterogeneity estimates are reported in Table S5. Quadratic sensitivity curves placed their minima at observed upper boundaries rather than producing stable interior turning points. This pattern is not evidence of a physiological optimum; it warns that a flexible polynomial can visually suggest curvature without sufficient dose support. Leave-one-study-out and high-dose-restriction analyses did not materially alter the overall conclusion, but boundary uncertainty remains greatest where dose observations are sparse.

The conversion to MET·min/week is both the enabling assumption and a principal source of uncertainty. Standard MET values describe population-average resting multiples and can misrepresent individual energetic demand when the resting metabolic rate is altered by age, sex, obesity, or disease. Resistance training is especially difficult because energy cost depends on exercise selection, load, repetitions, rest, tempo, and muscle mass, while HIIT alternates intensities within a session. The metric therefore standardizes estimated prescribed external exposure, not achieved individual dose or internal physiological load. The transparent assignment log and sensitivity models reduce hidden judgment but cannot eliminate exposure error. Non-differential dose error would generally flatten slopes; systematic differences by modality could alter comparative slopes.

The study population was deliberately broad, increasing relevance to general adult practice but complicating causal transport. Healthy workers; postmenopausal women; older adults; and people with diabetes, obesity, metabolic syndrome, hypertension, or fatty liver disease differ in hepatic triglyceride production, insulin resistance, medication use, and capacity to perform vigorous exercise. A common network effect assumes that relative treatment effects are exchangeable after accounting for measured differences. Although the network was connected and sensitivity results were stable, incomplete covariate reporting means that transitivity cannot be proven. The modality estimates should be regarded as average effects across the represented populations, not as personalized predictions. Recent syntheses report favorable lipid effects from concurrent exercise in people with type 2 diabetes [39,40], while a 2024 network meta-analysis found that lipid responses differed across training modalities in this population [41]. Such differences support clinical-status stratification in future dose–response trials.

Strengths include a broad multilingual search, explicit linkage of multiple reports from the same trial, duplicate screening and extraction, outcome-level RoB 2 assessment, harmonization of TG to mmol/L, a transparent MET-assignment hierarchy, multi-arm covariance structures, formal comparison of dose–response functions, prediction intervals, and structured certainty assessment.

The ranking analysis requires cautious interpretation. CE had the highest probability of being best (61.6%), followed by HIIT (38.2%); however, ranking probabilities do not incorporate certainty of evidence and can overstate distinctions when estimates overlap. The more defensible summary is estimate plus interval plus certainty: CE had the largest average network estimate, HIIT was promising but sparse, AE had the most extensive evidence, and RT remained uncertain.

Several limitations require emphasis. First, MET values standardize external exercise exposure but may not represent individualized energy cost, particularly in older or metabolically impaired adults. Second, intervention dose was often estimated from prescribed rather than achieved exercise, and adherence reporting was incomplete. Third, aggregate-data meta-regression is vulnerable to ecological bias and cannot fully separate baseline TG, weight change, diet, medication, and exercise effects. Fourth, change-score SDs and figure-derived values sometimes required imputation. Fifth, upper-bound dose estimates can be unstable in sparsely populated regions and depend on functional-form assumptions. Finally, broad clinical heterogeneity improves scope but may reduce transitivity; subgroup and sensitivity analyses should accompany the overall network.

Risk-of-bias sensitivity analysis was informative rather than merely reassuring. Excluding high-risk studies strengthened the pooled effect slightly and reduced τ from 0.106 to 0.037 mmol/L. This pattern suggests that methodological diversity—particularly incomplete randomization reporting, attrition, and selective availability of variance data—contributed materially to heterogeneity. It does not establish that lower-risk trials have a larger causal effect because the 26 retained studies may differ in population and intervention. Nevertheless, the result reinforces the need for transparent allocation, intention-to-treat analysis, and complete outcome reporting in exercise trials.

### 4.5. Research Implications

Achieved dose, not merely prescribed dose, should become a standard exercise-trial outcome. Attendance multiplied by planned minutes ignores reductions in intensity, incomplete sets, early session termination, and unsupervised non-adherence. Wearable heart-rate, accelerometry, or power data can quantify internal and external load, while session rating of perceived exertion provides a feasible complementary measure. Reporting distributions rather than group averages would enable adherence-adjusted estimands and dose–response analyses that respect within-arm exposure variation. Such data are especially important for HIIT, where the nominal intensity of work intervals may not describe the metabolic cost of warm-up, recovery, and cool-down.

Future exercise trials should report fasting status, assay procedures, baseline and follow-up TG means and SDs, within-group changes, adherence-adjusted and intention-to-treat estimates, adverse events, and detailed FITT parameters. Direct measurement of energy expenditure and reporting of achieved dose would reduce exposure misclassification. Trials comparing multiple randomized doses within the same modality are particularly valuable for identifying dose–response shape and should include sufficiently long follow-up to distinguish transient from sustained metabolic adaptation.

The small-study-effect signal also deserves a nuanced reading. The significant Egger intercept may indicate publication or selective-outcome bias, but it can also arise when smaller exercise trials enroll participants with higher baseline TGs, use more intensive supervision, or deliver more homogeneous protocols than larger pragmatic studies. Dependence among multiple contrasts further complicates the test. Accordingly, we did not apply trim-and-fill or use the regression as a correction. Prospective registration, protocol availability, and reporting of all prespecified lipid outcomes are more credible remedies than post hoc statistical adjustment.

## 5. Conclusions

Structured exercise was associated with lower circulating TGs, but the size and certainty of the average effect differed by modality. CE had the largest average network estimate, while HIIT and AE also produced favorable estimates and RT remained uncertain. Low or very low certainty, sparse direct comparisons, and prediction intervals preclude definitive claims of modality superiority. A modality-specific linear dose model best described the observed data, with no supported interior optimum or efficacy threshold. Lowest- and upper-observed-dose estimates should be treated as boundary descriptions and hypotheses for dose-ranging trials, not prescription targets. Clinical recommendations should integrate safety, fitness, adherence, comorbidity, achieved dose, and the broader benefits of physical activity.

## Figures and Tables

**Table 1:** Eligibility criteria for study inclusion and exclusion.

Domain	Inclusion Criteria	Exclusion Criteria
Population	Adults (≥18 years), irrespective of sex, baseline TG status, weight status, or cardiometabolic condition.	Children/adolescents; pregnancy; exclusively acute post-exercise studies.
Intervention	Structured AE, RT, CE, or HIIT lasting ≥2 weeks, with sufficient information to estimate weekly exercise dose.	Unstructured advice only; exercise combined with a co-intervention that differs between randomized groups and prevents isolation of the exercise effect.
Comparator	Non-exercise/usual-care control or another eligible exercise modality/dose.	No concurrent eligible comparator.
Outcome	Fasting or clearly defined plasma/serum TG reported as mean with variance at baseline and post intervention or as a change score.	TGs unavailable and cannot be derived; postprandial TGs only unless prespecified as a separate analysis.
Study design	Randomized controlled trials, including cluster and crossover trials when an appropriate randomized comparison can be extracted.	Non-randomized, quasi-randomized, uncontrolled, observational, or single-group studies.

**Table 2:** Characteristics of included study arms by exercise modality. Arm counts are descriptive randomized arms before the variance-based exclusion; dose is reported in MET·min/week.

Characteristic	AE	RT	CE	HIIT	CG
Arms	74	20	20	8	86
Participants	1777	329	473	121	1783
Dose	90–2835	315–1080	53–1800	660–1161	0
Duration, weeks	5–52	8–26	12–52	3–12	3–52

**Table 3:** Network meta-analysis estimates of triglyceride change by exercise modality versus control. Trial counts and analyzed arm-level contrasts are shown separately because multi-arm trials can contribute more than one contrast.

Comparison	MD	95% CI	95% Prediction Interval	Distinct Trials/Analyzed Contrasts
AE vs. CG	−0.130	−0.185 to −0.074	−0.312 to 0.053	52/74
CE vs. CG	−0.255	−0.355 to −0.156	−0.455 to −0.055	18/19
RT vs. CG	−0.057	−0.153 to 0.039	−0.255 to 0.141	18/20
HIIT vs. CG	−0.225	−0.392 to −0.059	−0.466 to 0.015	6/8

**Table 4:** Modality-specific linear dose–response estimates and fitted conditional mean effects at the upper observed dose boundaries. Observed dose is reported in MET·min/week.

Type	*n*	Observed Dose Range	Slope per 1000 MET·min/week	95% CI	Upper-Bound Effect (95% CI)
AE	74	90–2835	−0.128	−0.176 to −0.080	−0.363 (−0.499 to −0.227)
CE	19	53–1800	−0.307	−0.394 to −0.221	−0.553 (−0.709 to −0.398)
RT	20	315–1080	−0.081	−0.225 to 0.063	−0.087 (−0.243 to 0.068)
HIIT	8	660–1161	−0.239	−0.393 to −0.085	−0.278 (−0.456 to −0.099)

Note: *n* denotes analyzed arm-level contrasts. Negative values favor exercise. No supported interior optimum was identified. Upper-bound effects are conditional mean predictions from the fitted linear curves; they are not recommended doses, efficacy thresholds, or prediction intervals for a future study.

## Data Availability

Summary data supporting the findings of this study are provided in the article and Supplementary Materials. Additional data used in the analyses are available from the corresponding author upon reasonable request.

## Supplementary Materials

The following supporting information can be downloaded at: https://www.mdpi.com/article/10.3390/metabo16090683/s1: Table S1, database-specific search strategies; Table S2, PRISMA-NMA checklist; Table S3, characteristics of included independent trials; Table S4, trial-level risk-of-bias judgments; Table S5, dose–response model comparison; Table S6, pairwise random-effects analyses; Table S7, active–active network contrasts; Table S8, sensitivity analyses; Table S9, confidence assessment for modality-versus-control comparisons; Table S10, evidence-unit accounting and reconciliation; Figure S1, distribution of observed prescribed exercise dose (MET·min/week) by modality; Figure S2, trial-level risk-of-bias summary across RoB 2 domains and overall judgment; Figure S3, comparison-adjusted funnel plot for exercise versus control; Figure S4, overall quadratic dose–response sensitivity model; Figure S5, modality-specific quadratic sensitivity models; Figure S6, ranking probabilities from the network meta-analysis.
