# Supplementary material for: Dose–Response Relationships Between Exercise Modalities and Triglyceride Reduction in Adults: A Systematic Review and Model-Based Network Meta-Analysis
Source: Metabolites. 2026 Sep 17;16(9):683. doi: 10.3390/metabo16090683 (PMC13609431; doi:10.3390/metabo16090683)
Supplement: Supplementary file 1 [file metabolites-16-00683-s001.zip › metabolites-4528929-supplementary.pdf]

Supplementary Material

Table S1. Search Strategy and Reporting Items

Database	Core strategy framework
PubMed （3412）	("Exercise"[Mesh] OR "Exercise Therapy"[Mesh] OR exercise*[Title/Abstract] OR training[Title/Abstract] OR aerobic exercise*[Title/Abstract] OR aerobic training[Title/Abstract] OR resistance training[Title/Abstract] OR strength training[Title/Abstract] OR combined exercise[Title/Abstract] OR concurrent training[Title/Abstract] OR high intensity interval training[Title/Abstract] OR HIIT[Title/Abstract]) AND ("Triglycerides"[Mesh] OR triglyceride*[Title/Abstract] OR triacylglycerol*[Title/Abstract] OR TG[Title/Abstract]) AND (randomized controlled trial[Publication Type] OR randomized[Title/Abstract] OR randomised[Title/Abstract] OR randomly[Title/Abstract] OR trial[Title/Abstract])
Web of Science （4803）	TS=(exercise* OR "exercise training"OR "aerobic training" OR "resistance training" OR "strength training" OR "combined training" OR "concurrent training" OR "high intensity interval training" OR HIIT) AND TS= (triglyceride* OR triacylglycerol* OR TG) AND TS= (random* OR trial* OR "controlled trial")
Embase (370)	('exercise'/exp OR 'exercise therapy'/exp OR exercise*:ti,ab OR aerobic training:ti,ab OR resistance training:ti,ab OR strength training:ti,ab OR concurrent training:ti,ab OR high intensity interval training:ti,ab OR HIIT:ti,ab) AND ('triglyceride'/exp OR triglyceride*:ti,ab OR triacylglycerol*:ti,ab) AND (random*:ti,ab OR 'randomized controlled trial'/exp OR trial:ti,ab)
Scopus (5889)	TITLE-ABS-KEY (exercise* OR "exercise training" OR "aerobic training" OR "resistance training" OR "strength training" OR "combined training" OR "concurrent training" OR HIIT OR "high intensity interval training") AND TITLE-ABS-KEY (triglyceride* OR triacylglycerol* OR TG) AND TITLE-ABS-KEY (random* OR trial* OR "controlled trial")
Cochrane Library （3367）	(exercise* OR "exercise training" OR "physical activity" OR "aerobic exercise" OR "aerobic training" OR "resistance training" OR "strength training" OR "combined training" OR "concurrent training" OR "high intensity interval training" OR HIIT) AND (triglyceride* OR triacylglycerol* OR TG) AND (random* OR trial)

Table S2. PRISMA-NMA Checklist

Section/Topic	Item #	Checklist Item	Reported on Page/Section
TITLE			
Title	1	Identify the report as a systematic review incorporating a network meta-analysis (or related form of meta-analysis).	Title
ABSTRACT			
Structured summary	2	Provide a structured summary including, as applicable:<br>Background: main objectives<br>Methods: data sources; study eligibility criteria, participants, and interventions; study appraisal; and synthesis methods, such as network meta-analysis.<br>Results: number of studies and participants identified; summary estimates with corresponding confidence/credible intervals; treatment rankings may also be discussed. Authors may choose to summarize pairwise comparisons against a chosen treatment included in their analyses for brevity.<br>Discussion/Conclusions: limitations; conclusions and implications of findings.<br>Other: primary source of funding; systematic review registration number with registry name.	Abstract
INTRODUCTION			
Rationale	3	Describe the rationale for the review in the context of what is already known, including mention of why a network meta-analysis has been conducted.	Introduction
Objectives	4	Provide an explicit statement of questions being addressed, with reference to participants, interventions, comparisons, outcomes, and study design (PICOS).	Introduction (final paragraph)
METHODS			
Protocol and registration	5	Indicate whether a review protocol exists and if and where it can be accessed (e.g., Web address); and, if available, provide registration information, including registration number.	Methods opening paragraph; PROSPERO CRD420261469330
Eligibility criteria	6	Specify study characteristics (e.g., PICOS, length of follow-up) and report characteristics (e.g., years considered, language, publication status) used as criteria for eligibility, giving rationale. Clearly describe eligible treatments included in the treatment network, and note whether any have been clustered or merged into the same node (with justification).	Methods—2.1; Table 1
Information sources	7	Describe all information sources (e.g., databases with dates of coverage, contact with study authors to identify additional studies) in the search and date last searched.	Methods—2.2; Supplementary Appendix A
Search	8	Present full electronic search strategy for at least one database, including any limits used, such that it could be repeated.	Supplementary Appendix A

Section/Topic	Item #	Checklist Item	Reported on Page/Section
Study selection	9	State the process for selecting studies (i.e., screening, eligibility, included in systematic review, and, if applicable, included in the meta-analysis).	Results, Study Selection; Figure 1; Table S9
Data collection process	10	Describe method of data extraction from reports (e.g., piloted forms, independently, in duplicate) and any processes for obtaining and confirming data from investigators.	Methods—2.4
Data items	11	List and define all variables for which data were sought (e.g., PICOS, funding sources) and any assumptions and simplifications made.	Methods—2.4–2.5
Geometry of the network	S1	Describe methods used to explore the geometry of the treatment network under study and potential biases related to it. This should include how the evidence base has been graphically summarized for presentation, and what characteristics were compiled and used to describe the evidence base to readers.	Methods—2.8; Results—3.4; Figure 2
Risk of bias within individual studies	12	Describe methods used for assessing risk of bias of individual studies (including specification of whether this was done at the study or outcome level), and how this information is to be used in any data synthesis.	Methods—2.6
Summary measures	13	State the principal summary measures (e.g., risk ratio, difference in means). Also describe the use of additional summary measures assessed, such as treatment rankings and surface under the cumulative ranking curve (SUCRA) values, as well as modified approaches used to present summary findings from meta-analyses.	Methods—2.7–2.9
Planned methods of analysis	14	Describe the methods of handling data and combining results of studies for each network meta-analysis. This should include, but not be limited to:<br>Handling of multi-arm trials;<br>Selection of variance structure;<br>Selection of prior distributions in Bayesian analyses; and<br>Assessment of model fit.	Methods—2.8–2.9
Assessment of Inconsistency	S2	Describe the statistical methods used to evaluate the agreement of direct and indirect evidence in the treatment network(s) studied. Describe efforts taken to address its presence when found.	Methods—2.8–2.9
Risk of bias across studies	15	Specify any assessment of risk of bias that may affect the cumulative evidence (e.g., publication bias, selective reporting within studies).	Methods—2.6 and 2.8
Additional analyses	16	Describe methods of additional analyses if done, indicating which were pre-specified. This may include, but not be limited to, the following:<br>Sensitivity or subgroup analyses;<br>Meta-regression analyses;<br>Alternative formulations of the treatment network; and<br>Use of alternative prior distributions for Bayesian analyses (if applicable).	Methods—2.10
RESULTS†			
Study selection	17	Give numbers of studies screened, assessed for eligibility, and included in the review, with reasons for exclusions at each stage, ideally with a flow diagram.	Results, Study Selection; Figure 1; Table S9
Presentation of network structure	S3	Provide a network graph of the included studies to enable visualization of the geometry of the treatment network.	Results—3.4; Figure 2
Summary of network geometry	S4	Provide a brief overview of characteristics of the treatment network. This may include commentary on the abundance of trials and randomized patients for the different interventions and pairwise comparisons in the network, gaps of evidence in the treatment network, and potential biases reflected by the network structure.	Results—3.2 and 3.4; Table 2; Figure 2
Study characteristics	18	For each study, present characteristics for which data were extracted (e.g., study size, PICOS, follow-up period) and provide the citations.	Results—3.2; Table 2; Supplementary Table S2
Risk of bias within studies	19	Present data on risk of bias of each study and, if available, any outcome level assessment.	Results—3.3; Supplementary Table S3; Figure S2
Results of individual studies	20	For all outcomes considered (benefits or harms), present, for each study: 1) simple summary data for each intervention group, and 2) effect estimates and confidence intervals. Modified approaches may be needed to deal with information from larger networks.	Results; Tables 3–4 and S5–S7; Figures 3–4; Supplementary Data File 1 (Arm_Level and Contrast_Level)
Synthesis of results	21	Present results of each meta-analysis done, including confidence/credible intervals. In larger networks, authors may focus on comparisons versus a particular comparator (e.g. placebo or standard care), with full findings presented in an appendix. League tables and forest plots may be considered to summarize pairwise comparisons. If additional summary measures were explored (such as treatment rankings), these should also be presented.	Results—3.4–3.5; Tables 3–4; Figures 2–4
Exploration for inconsistency	S5	Describe results from investigations of inconsistency. This may include such information as measures of model fit to compare consistency and inconsistency models, P values from statistical tests, or summary of inconsistency estimates from different parts of the treatment network.	Results—3.4 and 3.7 (formal loop-based assessment limited by network geometry)
Risk of bias across studies	22	Present results of any assessment of risk of bias across studies for the evidence base being studied.	Results—3.6; Figure S3
Results of additional analyses	23	Give results of additional analyses, if done (e.g., sensitivity or subgroup analyses, meta-regression analyses, alternative network geometries studied, alternative choice of prior distributions for Bayesian analyses, and so forth).	Results—3.6; Supplementary Table S7
DISCUSSION			
Summary of evidence	24	Summarize the main findings, including the strength of evidence for each main outcome; consider their relevance to key groups (e.g., healthcare providers, users, and policy-makers).	Discussion—4.1–4.3; Supplementary Table S8
Limitations	25	Discuss limitations at study and outcome level (e.g., risk of bias), and at review level (e.g., incomplete retrieval of identified research, reporting bias). Comment on the validity of the assumptions, such as transitivity and consistency. Comment on any concerns regarding network geometry (e.g., avoidance of certain comparisons).	Discussion—4.4
Conclusions	26	Provide a general interpretation of the results in the context of other evidence, and implications for future research.	Discussion—4.5; Conclusions
FUNDING			
Funding	27	Describe sources of funding for the systematic review and other support (e.g., supply of data); role of funders for the systematic review. This should also include information regarding whether funding has been received from manufacturers of treatments in the network and/or whether some of the authors are content experts with professional conflicts of interest that could affect use of treatments in the network.	Funding; Conflicts of Interest

Note. Checklist wording follows the PRISMA extension for network meta-analyses (PRISMA-NMA). Reporting locations refer to the accompanying manuscript and this supplement. The review was registered in PROSPERO (CRD420261469330), with a prespecified minimum intervention duration of 2 weeks.

Abbreviation: PICOS, population, intervention, comparators, outcomes, and study design.

Table S3. Characteristics of included independent trials.

Author/year	Population	N	Arms	Weeks	Dose range	RoB
Fang et al. 2019	Adults; Overweight; Employees/workers	75	CG (1), AE (1)	12–12	990–990	High risk
Sillanpaa et al. 2009	Women; Older adults; Healthy	61	CE (1), CG (1), AE (1), RT (1)	21–21	525–1275	Some concerns
O'Donovan et al. 2005	Men; Middle-aged; Sedentary/inactive	42	AE (2), CG (1)	24–24	1080–1200	Some concerns
Trajkovic et al. 2020	Men; Middle-aged; Type 2 diabetes; Hypertension; Obesity	26	CG (1), AE (1)	10–10	900–900	High risk
Abdelbasset et al. 2019	Women; NAFLD; Obesity	32	CG (1), HIIT (1)	8–8	1003–1003	Some concerns
Afzalpour et al. 2008	Men; Sedentary/inactive; Healthy	44	AE (2), CG (1)	8–8	844–956	High risk
Agner et al. 2018	Women; Older adults; Metabolic syndrome; Dyslipidemia; Obesity	41	CE (1), CG (1)	12–12	900–900	Some concerns
Arca et al. 2014	Postmenopausal women; Older adults; Hypertension	52	AE (2), CG (1)	12–12	825–900	High risk
Babu et al. 2022	Women; Type 2 diabetes; NAFLD; Metabolic syndrome; Dyslipidemia; Obesity; Sedentary/inacti	42	CG (1), HIIT (1)	12–12	792–792	Some concerns
Baker et al. 1986	Men; Middle-aged; Obesity; Sedentary/inactive	34	AE (1), CG (1)	20–20	547–547	High risk
Binder et al. 1996	Postmenopausal women; Older adults; Healthy	71	CG (2), AE (2)	48–48	789–789	High risk
Biteli et al. 2021	Postmenopausal women; Type 2 diabetes; Metabolic syndrome; Dyslipidemia; Obesity	70	CG (2), CE (2)	20–20	855–855	Some concerns
Boardley et al. 2007	Adults; Older adults; Obesity; Sedentary/inactive	131	AE (1), CE (1), CG (1), RT (1)	16–16	300–660	High risk
Bobeuf et al. 2011	Women; Older adults	57	RT (2), CG (2)	26–26	1080–1080	High risk
Bock et al. 2019	Women; Sedentary/inactive; Healthy	283	AE (2), CG (1)	12–12	750–1095	Some concerns
Bonfante et al. 2017	Men; Obesity	22	CE (1), CG (1)	24–24	900–900	High risk
Bonfante et al. 2022	Adults; Type 2 diabetes; Obesity	34	CE (1), CG (1)	16–16	1125–1125	High risk
Boyden et al. 1993	Women; Sedentary/inactive; Healthy	88	CG (1), RT (1)	22–22	315–315	High risk
Busby et al. 1985	Adults	44	CG (2), AE (2)	12–12	1095–1095	High risk

Cho et al. 2011	Women; Metabolic syndrome; Hypertension; Dyslipidemia; Obesity	35	AE (2), CG (1)	12–12	540–990	High risk
Colado et al. 2009	Postmenopausal women; Middle-aged; Hypertension; Obesity; Sedentary/inactive	46	RT (2), CG (1)	24–24	315–342	High risk
Dalleck et al. 2009	Postmenopausal women; Type 2 diabetes; Obesity; Sedentary/inactive	26	AE (2), CG (1)	12–12	825–1238	High risk
Sunami et al. 1999	Women; Older adults; Sedentary/inactive	40	CG (1), AE (1)	22–22	1152–1152	Some concerns
Eguchi et al. 2012	Women; Older adults; Employees/workers	28	AE (2), CG (1)	12–12	540–720	High risk
Elliott et al. 2002	Postmenopausal women; Older adults; Sedentary/inactive	15	CG (1), RT (1)	8–8	810–810	High risk
Taniguchi et al. 2016	Men; Older adults; Obesity	54	CG (1), AE (1)	5–5	936–936	High risk
Fahlman et al. 2002	Women; Older adults; Healthy	45	AE (1), CG (1), RT (1)	10–10	570–900	Some concerns
Fenkci et al. 2006	Women; Type 2 diabetes; Obesity	51	AE (1), CG (1), RT (1)	12–12	547–810	Some concerns
Ha et al. 2018	Women; Older adults	40	AE (2), CG (2)	12–12	990–990	Some concerns
Hagan et al. 1986	Adults	96	CG (4), AE (4)	12–12	570–570	High risk
Hallsworth et al. 2015	Adults; Type 2 diabetes; NAFLD; Obesity	23	CG (1), HIIT (1)	12–12	924–924	High risk
Hersey et al. 1994	Women; Older adults; Dyslipidemia	42	CG (1), AE (1), RT (1)	26–26	420–876	High risk
Hornstrup et al. 2019	Men; Young adults; Sedentary/inactive	26	CG (1), AE (1)	12–12	927–927	High risk
Houghton et al. 2017	Adults	24	CE (1), CG (1)	12–12	945–945	Some concerns
Hsieh et al. 2016	Adults; Older adults; Type 2 diabetes; Obesity	29	RT (1), CG (1)	12–12	610–610	Some concerns
Huttunen et al. 1979	Men; Middle-aged; Hypertension; Sedentary/inactive	90	CG (1), AE (1)	16–16	732–732	High risk
Vicente-Campos et al. 2012	Women; Older adults; Sedentary/inactive	43	CG (1), CE (1)	30–30	875–875	Some concerns
Jennings et al. 2015	Adults	70	AE (1), CG (1)	12–12	912–912	Some concerns
Duan & Lu 2024	Women; Type 2 diabetes; Obesity; Sedentary/inactive	36	AE (1), CG (1), RT (1)	12–12	648–810	Some concerns
Juneau et al. 1987	Women; Middle-aged; Overweight; Sedentary/inactive; Employees/workers	113	CG (2), AE (2)	24–24	1292–1485	High risk
Kantyka et al. 2015	Postmenopausal women; Older adults; Obesity; Sedentary/inactive	21	AE (1), CG (1)	12–12	742–742	High risk

Kraus et al. 2002	Women; Dyslipidemia; Obesity; Sedentary/inactive	101	AE (3), CG (1)	32–32	765–1020	High risk
LeMura et al. 2000	Women; Young adults; Sedentary/inactive	44	AE (1), CE (1), CG (1), RT (1)	16–16	525–825	High risk
Liao et al. 2015	Adults	108	CG (1), AE (1)	12–12	825–825	High risk
Libardi et al. 2012	Men; Older adults; Type 2 diabetes; Hypertension; Dyslipidemia; Sedentary/inactive	47	CE (1), CG (1), AE (1), RT (1)	16–16	630–990	High risk
Manning et al. 1991	Adults	22	CG (1), RT (1)	12–12	720–720	High risk
Mendham et al. 2014	Men; Type 2 diabetes; Sedentary/inactive	26	CG (1), CE (1)	12–12	1350–1350	Some concerns
Morey et al. 2012	Women; Older adults; Type 2 diabetes; Obesity	302	AE (1), CG (1)	52–52	825–825	Some concerns
Motoyama et al. 1995	Women; Older adults; Hypertension; Sedentary/inactive	30	CG (1), AE (1)	39–39	270–270	High risk
Murphy et al. 2006	Women; Sedentary/inactive	20	CG (1), AE (1)	8–8	432–432	High risk
Murtagh et al. 2005	Women; Sedentary/inactive ; Healthy	32	AE (2), CG (1)	12–12	288–288	High risk
Niederseer et al. 2011	Women; Older adults; Obesity; Sedentary/inactive	34	AE (1), CG (1)	12–12	2835–2835	High risk
Nieman et al. 2002	Women; Obesity	91	CG (2), AE (2)	12–12	684–684	High risk
Nualnim et al. 2012	Women; Older adults; Hypertension; Obesity	43	CG (1), AE (1)	12–12	783–783	High risk
Park et al. 2003	Women; Middle-aged; Hypertension; Obesity	30	AE (1), CE (1), CG (1)	24–24	1800–2628	High risk
Park et al. 2014	Adults; Older adults; Type 2 diabetes	20	CE (1), CG (1)	12–12	750–750	Some concerns
Patterson et al. 2016	Women; Sedentary/inactive	33	AE (2), CG (1)	8–8	1326–1326	High risk
Prabhakaran et al. 1999	Women; Dyslipidemia; Sedentary/inactive	24	CG (1), RT (1)	14–14	900–900	High risk
Raz et al. 1988	Men; Young adults; Obesity; Sedentary/inactive	55	AE (1), CG (1)	9–9	742–742	High risk
Rowland et al. 2018	Women; Older adults; Sedentary/inactive ; Employees/workers	40	CG (1), AE (1)	12–12	685–685	Some concerns
Herawati et al. 2025	Women; Middle-aged; Hypertension	87	HIIT (2), AE (1), CG (1)	10–10	90–792	Some concerns
Schuit et al. 1998	Women; Older adults; Hypertension; Obesity; Sedentary/inactive	176	AE (2), CG (1)	26–26	866–945	Some concerns
Smith-Ryan et al. 2015	Men; Hypertension; Obesity	25	HIIT (2), CG (1)	3–3	660–660	Some concerns
Sousa et al. 2014	Men; Older adults; Dyslipidemia; Obesity	48	AE (1), CG (1), CE (1)	32–32	1314–1350	Some concerns

Stensvold et al. 2010	Men and women with metabolic syndrome	40	CG (1), HIIT (1), CE (1), RT (1)	12–12	525–1161	Some concerns
Suter et al. 1994	Sedentary, nonsmoking, apparently healthy men	75	AE (2), CG (1)	26–26	630–1080	High risk
Takeshima et al. 2004	Sedentary, apparently healthy older men and women	35	CE (1), CG (1)	12–12	525–525	High risk
Ho et al. 2012	Overweight or obese adults	64	CG (1), AE (1), RT (1), CE (1)	12–12	350–495	High risk
Thompson et al. 2010	Sedentary middle-aged men	41	CG (1), AE (1)	24–24	1540–1540	High risk
Tokudome et al. 2004	Community-dwelling older adults; predominantly women	230	CE (1), CG (1)	12–12	53–53	High risk
Vinetti et al. 2015	Men with type 2 diabetes, metabolic syndrome, overweight/grade I obesity	20	CE (1), CG (1)	52–52	600–600	High risk
Wen et al. 2016	Postmenopausal women with low bone mass	46	AE (1), CG (1)	10–10	1620–1620	High risk
Kim et al. 2016	Older adults with hypertension	50	AE (1), CG (1)	12–12	900–900	High risk
Woolf-May et al. 1998	Low-active middle-aged and older men and women	46	AE (2), CG (1)	18–18	520–560	High risk
Wooten et al. 2011	Sedentary obese postmenopausal women not using hormone replacement therapy	21	CG (1), RT (1)	12–12	515–515	High risk
Wu et al. 2011	Middle-aged adults at elevated risk of type 2 diabetes	135	CG (1), AE (1)	39–39	825–825	Some concerns

Table S4. Trial-level risk-of-bias judgments.

Author/year	D1	D2	D3	D4	D5	Overall
Fang et al. 2019	High risk	Some concerns	High risk	Low risk	Low risk	High risk
Sillanpaa et al. 2009	Some concerns	Some concerns	Some concerns	Low risk	Low risk	Some concerns
O'Donovan et al. 2005	Some concerns	Some concerns	Some concerns	Low risk	Low risk	Some concerns
Trajkovic et al. 2020	High risk	Some concerns	Low risk	Low risk	Low risk	High risk
Abdelbasset et al. 2019	Low risk	Some concerns	Low risk	Low risk	Low risk	Some concerns
Afzalpour et al. 2008	High risk	Some concerns	High risk	Low risk	Low risk	High risk
Agner et al. 2018	Some concerns	Some concerns	Some concerns	Low risk	Low risk	Some concerns
Arca et al. 2014	High risk	Some concerns	Low risk	Low risk	Low risk	High risk
Babu et al. 2022	Some concerns	Some concerns	Some concerns	Low risk	Low risk	Some concerns
Baker et al. 1986	High risk	Some concerns	Some concerns	Low risk	Low risk	High risk
Binder et al. 1996	High risk	Some concerns	Low risk	Low risk	Low risk	High risk
Biteli et al. 2021	Some concerns	Some concerns	Some concerns	Low risk	Low risk	Some concerns
Boardley et al. 2007	High risk	Some concerns	Some concerns	Low risk	Low risk	High risk

Bobeuf et al. 2011	High risk	Some concerns	Low risk	Low risk	Low risk	High risk
Bock et al. 2019	Some concerns	Some concerns	Some concerns	Low risk	Low risk	Some concerns
Bonfante et al. 2017	High risk	Some concerns	Low risk	Low risk	Low risk	High risk
Bonfante et al. 2022	High risk	Some concerns	Low risk	Low risk	Low risk	High risk
Boyden et al. 1993	High risk	Some concerns	High risk	Low risk	Low risk	High risk
Busby et al. 1985	High risk	Some concerns	Some concerns	Low risk	Low risk	High risk
Cho et al. 2011	Low risk	Some concerns	High risk	Low risk	Low risk	High risk
Colado et al. 2009	High risk	Some concerns	High risk	Low risk	Low risk	High risk
Dalleck et al. 2009	High risk	Some concerns	Some concerns	Low risk	Low risk	High risk
Sunami et al. 1999	Some concerns	Some concerns	Some concerns	Low risk	Low risk	Some concerns
Eguchi et al. 2012	High risk	Some concerns	Some concerns	Low risk	Low risk	High risk
Elliott et al. 2002	High risk	Some concerns	Low risk	Low risk	Low risk	High risk
Taniguchi et al. 2016	High risk	Some concerns	Low risk	Low risk	Low risk	High risk
Fahlman et al. 2002	Some concerns	Some concerns	Some concerns	Low risk	Low risk	Some concerns
Fenkci et al. 2006	Some concerns	Some concerns	Some concerns	Low risk	Low risk	Some concerns
Ha et al. 2018	Some concerns	Some concerns	Some concerns	Low risk	Low risk	Some concerns
Hagan et al. 1986	High risk	Some concerns	High risk	Low risk	Low risk	High risk
Hallsworth et al. 2015	High risk	Some concerns	Low risk	Low risk	Low risk	High risk
Hersey et al. 1994	High risk	Some concerns	High risk	Low risk	Low risk	High risk
Hornstrup et al. 2019	High risk	Some concerns	High risk	Low risk	Low risk	High risk
Houghton et al. 2017	Some concerns	Some concerns	Some concerns	Low risk	Low risk	Some concerns
Hsieh et al. 2016	Some concerns	Some concerns	Low risk	Low risk	Low risk	Some concerns
Huttunen et al. 1979	High risk	Some concerns	Some concerns	Low risk	Low risk	High risk
Vicente-Campos et al. 2012	Some concerns	Some concerns	Some concerns	Low risk	Low risk	Some concerns
Jennings et al. 2015	Some concerns	Some concerns	Low risk	Low risk	Low risk	Some concerns
Duan & Lu 2024	Some concerns	Some concerns	Some concerns	Low risk	Low risk	Some concerns
Juneau et al. 1987	High risk	Some concerns	Some concerns	Low risk	Low risk	High risk
Kantyka et al. 2015	High risk	Some concerns	High risk	Low risk	Low risk	High risk
Kraus et al. 2002	High risk	Some concerns	Some concerns	Low risk	Low risk	High risk
LeMura et al. 2000	High risk	Low risk	Some concerns	Low risk	Low risk	High risk
Liao et al. 2015	High risk	Some concerns	Some concerns	Low risk	Low risk	High risk
Libardi et al. 2012	High risk	Some concerns	Low risk	Low risk	Low risk	High risk
Manning et al. 1991	High risk	Some concerns	Some concerns	Low risk	Low risk	High risk
Mendham et al. 2014	Some concerns	Some concerns	Some concerns	Low risk	Low risk	Some concerns
Morey et al. 2012	Some concerns	Some concerns	Low risk	Low risk	Low risk	Some concerns
Motoyama et al. 1995	High risk	Some concerns	High risk	Low risk	Low risk	High risk
Murphy et al. 2006	High risk	Low risk	Some concerns	Low risk	Low risk	High risk
Murtagh et al. 2005	High risk	Some concerns	Some concerns	Low risk	Some concerns	High risk
Niederseer et al. 2011	High risk	Some concerns	Some concerns	Low risk	Low risk	High risk

Nieman et al. 2002	High risk	Some concerns	Some concerns	Low risk	Low risk	High risk
Nualnim et al. 2012	High risk	Some concerns	Some concerns	Low risk	Some concerns	High risk
Park et al. 2003	High risk	Some concerns	High risk	Low risk	Low risk	High risk
Park et al. 2014	Some concerns	Some concerns	Some concerns	Low risk	Low risk	Some concerns
Patterson et al. 2016	High risk	Some concerns	Some concerns	Low risk	Some concerns	High risk
Prabhakaran et al. 1999	High risk	Some concerns	Some concerns	Low risk	Low risk	High risk
Raz et al. 1988	High risk	Low risk	Some concerns	Low risk	Low risk	High risk
Rowland et al. 2018	Some concerns	Some concerns	Low risk	Low risk	Some concerns	Some concerns
Herawati et al. 2025	Some concerns	Some concerns	Some concerns	Low risk	Low risk	Some concerns
Schuit et al. 1998	Some concerns	Some concerns	Some concerns	Low risk	Low risk	Some concerns
Smith-Ryan et al. 2015	Some concerns	Some concerns	Some concerns	Low risk	Low risk	Some concerns
Sousa et al. 2014	Some concerns	Some concerns	Some concerns	Low risk	Low risk	Some concerns
Stensvold et al. 2010	Low risk	Some concerns	Some concerns	Low risk	Low risk	Some concerns
Suter et al. 1994	High risk	Some concerns	Low risk	Low risk	Low risk	High risk
Takeshima et al. 2004	High risk	Some concerns	Low risk	Low risk	Low risk	High risk
Ho et al. 2012	Low risk	Some concerns	High risk	Low risk	Low risk	High risk
Thompson et al. 2010	Some concerns	Some concerns	High risk	Low risk	Low risk	High risk
Tokudome et al. 2004	High risk	Low risk	Some concerns	Low risk	Some concerns	High risk
Vinetti et al. 2015	High risk	Some concerns	Low risk	Low risk	Low risk	High risk
Wen et al. 2016	High risk	Some concerns	Some concerns	Low risk	Low risk	High risk
Kim et al. 2016	High risk	Some concerns	High risk	Low risk	Low risk	High risk
Woolf-May et al. 1998	High risk	Some concerns	High risk	Low risk	Some concerns	High risk
Wooten et al. 2011	High risk	Some concerns	Some concerns	Low risk	Some concerns	High risk
Wu et al. 2011	Some concerns	Some concerns	Low risk	Low risk	Low risk	Some concerns

Figure S1. Distribution of observed prescribed exercise dose (MET·min/week) by modality. Points represent randomized exercise arms; distributions are restricted to observed data and should not be interpreted as recommended ranges.

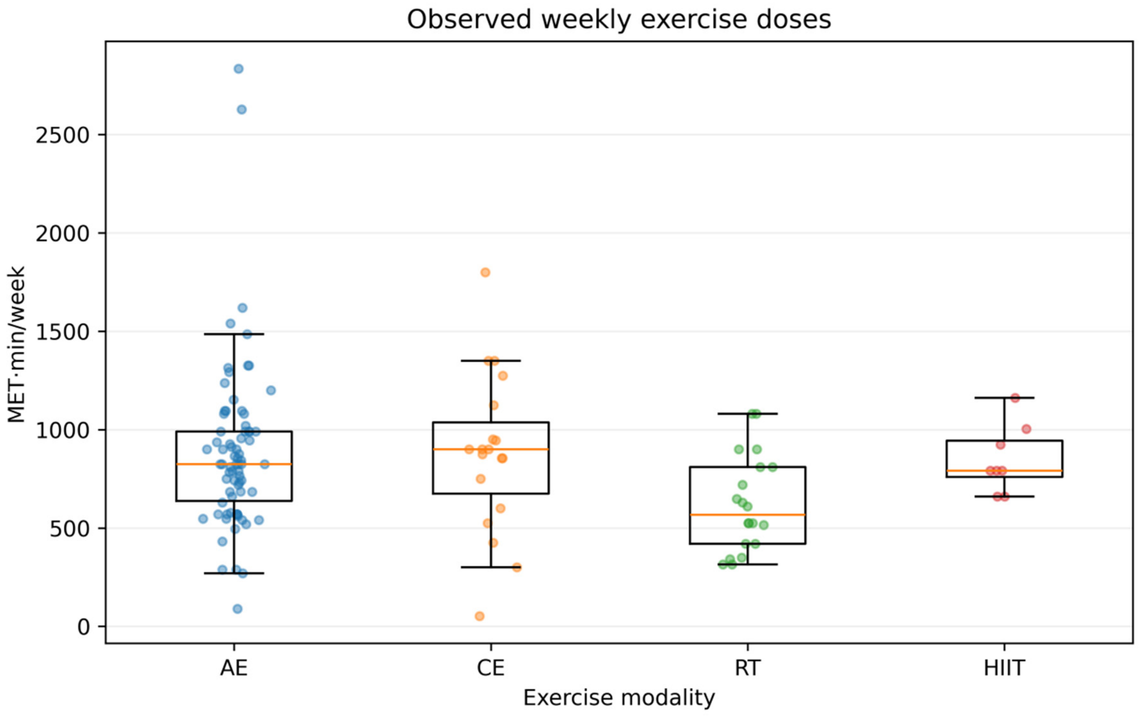

Figure S2. Trial-level risk-of-bias summary across RoB 2 domains and overall judgment.

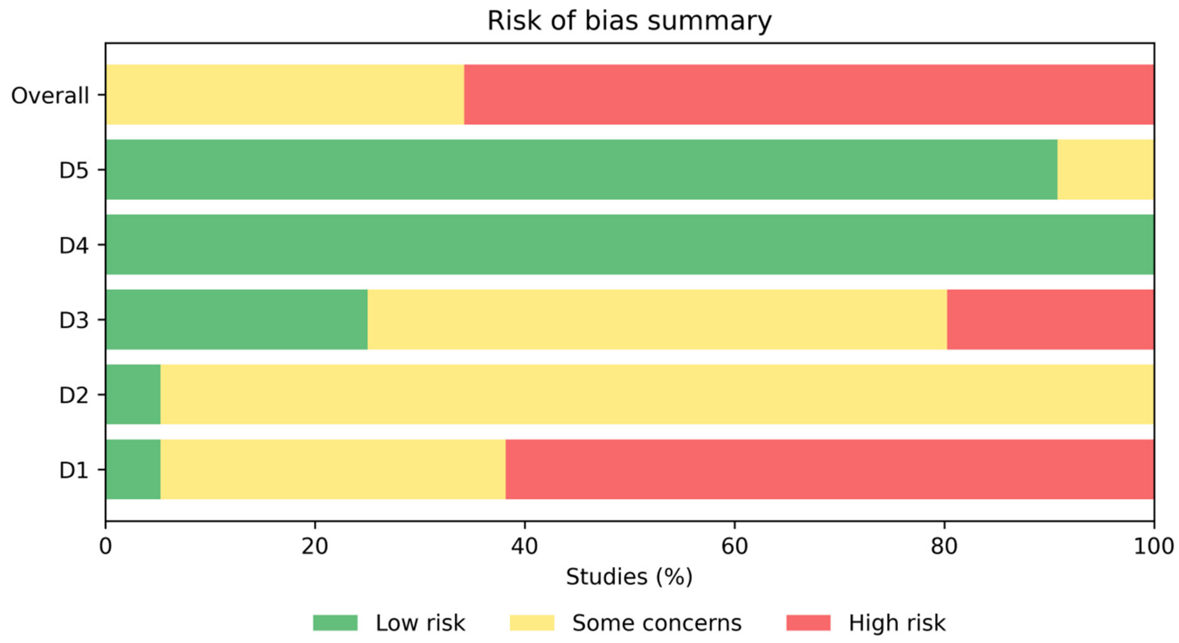

Figure S3. Comparison-adjusted funnel plot for exercise versus control. The plot is exploratory because multiple contrasts from the same trial are correlated.

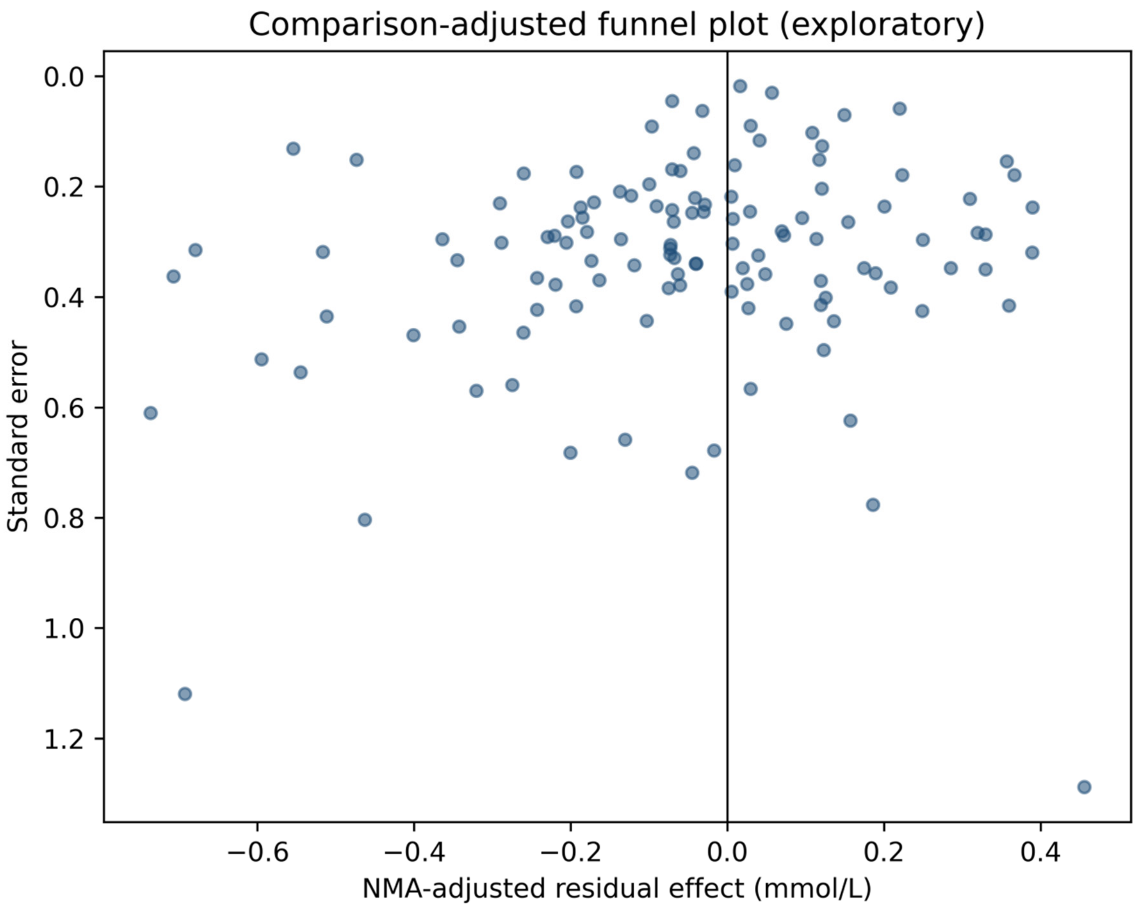

Table S5. Dose–response model comparison. Parameters are the number of dose-function coefficients; τ is residual between-study heterogeneity. Model choice was based primarily on AICc, supported by log likelihood, AIC, residual heterogeneity, and graphical fit.

Model	Parameters	τ	Log likelihood	AIC	AICc
modality_linear	4	0.065	5.278	-0.56	-0.03
modality_quadratic	8	0.052	7.077	3.85	5.47
common_linear	1	0.102	-3.095	10.19	10.29
common_quadratic	2	0.097	-2.063	10.13	10.33

Figure S4. Overall quadratic dose–response sensitivity model. The curve is constrained through the control dose; shaded bands show uncertainty in the fitted mean. Boundary behavior is not evidence of a physiological optimum.

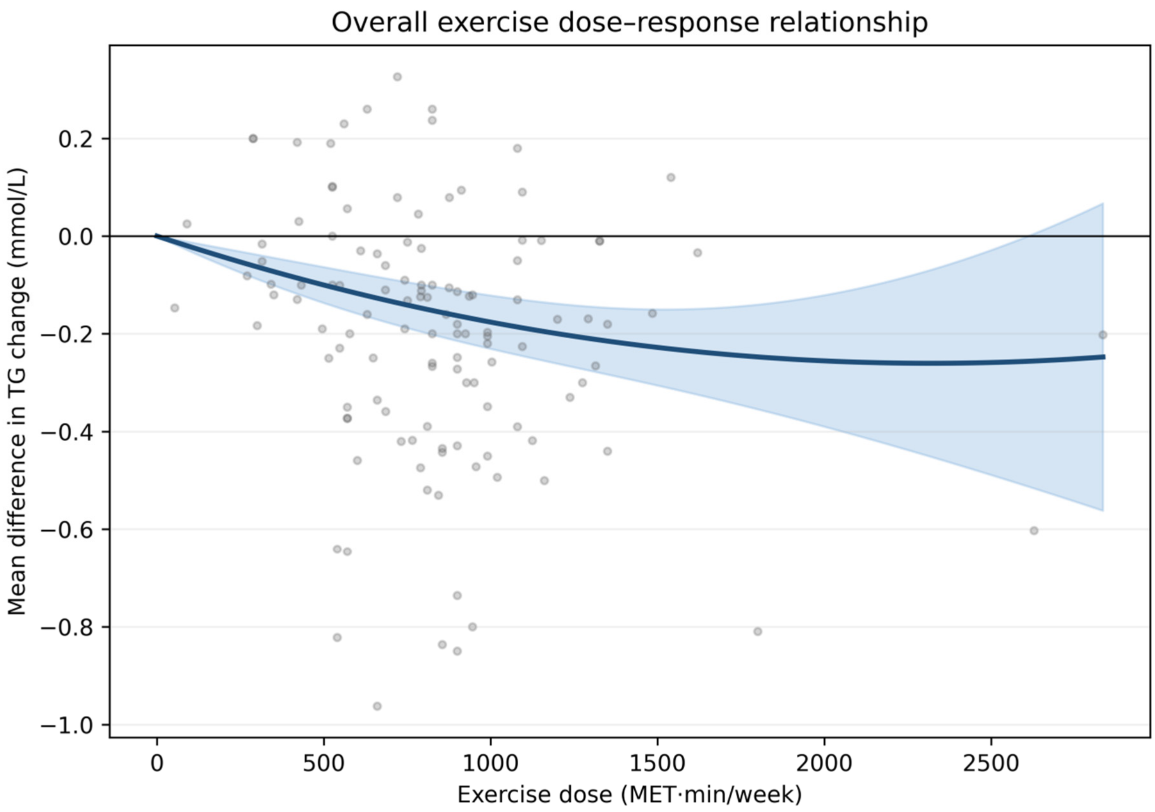

Figure S5. Modality-specific quadratic sensitivity models. Curves are shown only within observed modality-specific dose ranges; sparse boundary regions and unstable turning points require caution.

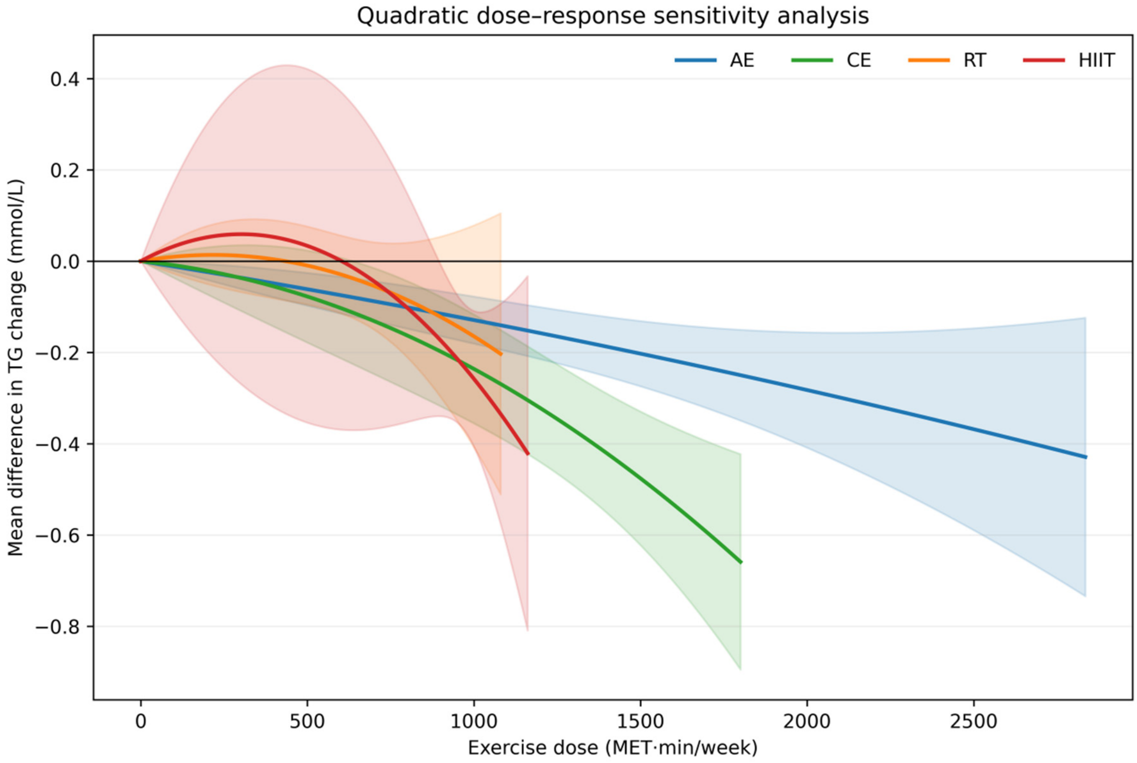

Figure S6. Ranking probabilities from the network meta-analysis. Rankings are secondary summaries and do not incorporate certainty of evidence; they should not be interpreted as proof of superiority.

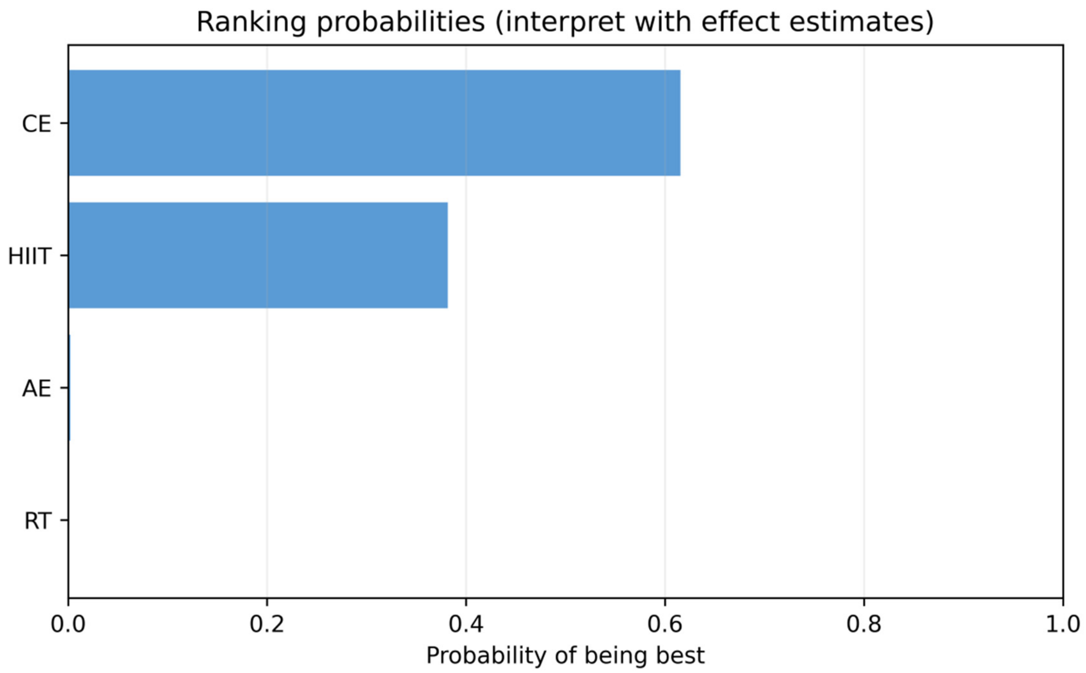

Table S6. Pairwise random-effects analyses. Trial counts and analyzed arm-level contrasts are reported separately.

Comparison	MD (95% CI)	95% prediction interval	τ	Distinct trials / analyzed contrasts
AE vs CG	-0.138 (-0.195 to -0.082)	-0.318 to 0.041	0.087	52 / 74
CE vs CG	-0.310 (-0.466 to -0.154)	-0.751 to 0.131	0.210	18 / 19
RT vs CG	-0.055 (-0.133 to 0.024)	-0.174 to 0.065	0.046	18 / 20
HIIT vs CG	-0.239 (-0.352 to -0.125)	-0.352 to -0.125	0.000	6 / 8

Table S7. Active–active network contrasts. Estimates are row modality minus comparator modality; negative values favor the row modality. Confidence intervals, not point estimates alone, should guide interpretation.

Comparison	MD (95% CI), mmol/L
AE vs CE	0.126 (0.018 to 0.234)
AE vs RT	-0.073 (-0.175 to 0.030)
AE vs HIIT	0.096 (-0.078 to 0.269)
CE vs RT	-0.198 (-0.331 to -0.065)
CE vs HIIT	-0.030 (-0.223 to 0.163)
RT vs HIIT	0.168 (-0.023 to 0.360)

Table S8. Sensitivity analyses. Counts distinguish independent trials from analyzed arm-level contrasts.

Analysis	MD (95% CI)	95% prediction interval	τ	Studies	Contrasts
Primary	-0.149 (-0.199 to -0.099)	-0.362 to 0.064	0.106	76	121
Exclude high risk	-0.167 (-0.234 to -0.100)	-0.265 to -0.069	0.037	26	42
Duration >=8 weeks	-0.149 (-0.200 to -0.099)	-0.364 to 0.065	0.106	74	118
Dose <=2000 MET-min/week	-0.149 (-0.200 to -0.099)	-0.363 to 0.064	0.106	75	119

Table S9. Confidence assessment for modality versus control comparisons.

Comparison	Confidence	Main reasons
AE vs CG	Low	Major within-study concerns; possible reporting bias; prediction interval crosses null.
CE vs CG	Low	Major within-study concerns and heterogeneity; favorable prediction interval.
HIIT vs CG	Low	Sparse evidence; imprecision and prediction interval crossing null.
RT vs CG	Very low	Imprecise effect and slope; high risk of bias.

Abbreviations and analytic units. AE, aerobic exercise; CE, combined aerobic and resistance exercise; CG, control group; HIIT, high-intensity interval training; MBNMA, model-based network meta-analysis; NMA, network meta-analysis; RT, resistance training. An arm-level contrast compares one randomized active arm with its matched control. A comparator block comprises one independently randomized control and its eligible active arms. A network edge label counts one unique modality-by-comparator-block link and therefore differs from both trial and arm-level-contrast counts.

Table S10. Evidence-unit accounting and reconciliation.

Analysis stage	Unit	Count	Definition/reconciliation

Study selection	Eligible reports	121	Reports were linked to their underlying trials before analysis.
Study selection	Independent randomized trials	76	The primary study unit after report linkage.
Dataset	Randomized arms	208	122 active exercise arms plus 86 control arms.
Contrast construction	Derived active–control contrasts	122	One contrast per active arm against its matched control.
Quantitative exclusion	Zero-variance contrast	1	LeMura et al. (2000), CE versus CG; excluded from both NMA and MBNMA.
Principal NMA/MBNMA	Analyzed arm-level contrasts	121	All remaining active–control contrasts.
Network covariance	Independent comparator blocks	86	One independently randomized control arm defines one block.
Figure 2 network	Unique modality–block links	104	AE 60, CE 19, RT 19, HIIT 6; repeated same-modality arms sharing one control count once.
Table 3	Distinct trials by modality	—	AE 52, CE 18, RT 18, HIIT 6; totals are non-additive because multi-modality trials contribute to more than one category.

Note. Supplementary Data File 1 provides the complete arm-level source data, explicit active-to-control mapping, formula-derived contrasts, inclusion/exclusion flags, and these accounting totals.

Reference

1. Fang, Y. Y., Huang, C. Y., & Hsu, M. C. (2019). Effectiveness of a physical activity program on weight, physical fitness, occupational stress, job satisfaction and quality of life of overweight employees in high-tech industries: A randomized controlled study. International Journal of Occupational Safety and Ergonomics, 25(4), 621–629. https://doi.org/10.1080/10803548.2018.1438839

2. Sillanpää, E., Laaksonen, D. E., Häkkinen, A., Karavirta, L., Jensen, B., Kraemer, W. J., Nyman, K., & Häkkinen, K. (2009). Body composition, fitness, and metabolic health during strength and endurance training and their combination in middle-aged and older women. European Journal of Applied Physiology, 106(2), 285–296. https://doi.org/10.1007/s00421-009-1013-x

3. O'Donovan, G., Owen, A., Bird, S. R., Kearney, E. M., Nevill, A. M., Jones, D. W., & Woolf-May, K. (2005). Changes in cardiorespiratory fitness and coronary heart disease risk factors following 24 weeks of moderate- or high-intensity exercise of equal energy cost. Journal of Applied Physiology, 98(5), 1619–1625. https://doi.org/10.1152/japplphysiol.01310.2004

4. Trajković, N., Sporiš, G., Krističević, T., & Bogataj, Š. (2020). Effects of small-sided recreational volleyball on health markers and physical fitness in middle-aged men. International Journal of Environmental Research and Public Health, 17(9), 3021. https://doi.org/10.3390/ijerph17093021

5. Abdelbasset, W. K., Tantawy, S. A., Kamel, D. M., Alqahtani, B. A., & Soliman, G. S. (2019). A randomized controlled trial on the effectiveness of 8-week high-intensity interval exercise on intrahepatic triglycerides, visceral lipids, and health-related quality of life in diabetic obese patients with nonalcoholic fatty liver disease. Medicine, 98(12), e14918. https://doi.org/10.1097/MD.0000000000014918

6. Afzalpour, M. E., Gharakhanlou, R., Gaeini, A. A., Mohebbi, H., Hedayati, M., & Khazaei, M. (2008). The effects of aerobic exercises on the serum oxidized LDL and total antioxidant capacity in non-active men. CVD Prevention and Control, 3(2), 77–82. https://doi.org/10.1016/j.cvdpc.2008.01.002

7. Agner, V. F. C., Garcia, M. C., Taffarel, A. A., Mourão, C. B., da Silva, I. P., da Silva, S. P., Peccin, M. S., & Lombardi, I., Jr. (2018). Effects of concurrent training on muscle strength in older adults with metabolic syndrome: A randomized controlled clinical trial. Archives of Gerontology and Geriatrics, 75, 158–164. https://doi.org/10.1016/j.archger.2017.12.011

8. Arca, E. A., Martinelli, B., Martin, L. C., Waisberg, C. B., & Franco, R. J. D. S. (2014). Aquatic exercise is as effective as dry land training for blood pressure reduction in postmenopausal hypertensive women. Physiotherapy Research International, 19(2), 93–98. https://doi.org/10.1002/pri.1565

9. Babu, A. F., Csader, S., Männistö, V., Tauriainen, M. M., Pentikäinen, H., Savonen, K., Klåvus, A., Koistinen, V., Hanhineva, K., & Schwab, U. (2022). Effects of exercise on NAFLD using non-targeted metabolomics in adipose tissue, plasma, urine, and stool. Scientific Reports, 12(1), 6485. https://doi.org/10.1038/s41598-022-10481-9

10. Baker, T. T., Allen, D., Lei, K. Y., & Willcox, K. K. (1986). Alterations in lipid and protein profiles of plasma lipoproteins in middle-aged men consequent to an aerobic exercise program. Metabolism, 35(11), 1037–1043. https://doi.org/10.1016/0026-0495(86)90040-5

11. Binder, E. F., Birge, S. J., & Kohrt, W. M. (1996). Effects of endurance exercise and hormone replacement therapy on serum lipids in older women. Journal of the American Geriatrics Society, 44(3), 231–236. https://doi.org/10.1111/j.1532-5415.1996.tb00907.x

12. Biteli, P., Barbalho, S. M., Detregiachi, C. R. P., dos Santos Haber, J. F., & Chagas, E. F. B. (2021). Dyslipidemia influences the effect of physical exercise on inflammatory markers in obese postmenopausal women: A randomized clinical trial. Experimental Gerontology, 150, 111355. https://doi.org/10.1016/j.exger.2021.111355

13. Boardley, D., Fahlman, M., Topp, R., Morgan, A. L., & McNevin, N. (2007). The impact of exercise training on blood lipids in older adults. The American Journal of Geriatric Cardiology, 16(1), 30–35. https://doi.org/10.1111/j.1076-7460.2007.05353.x

14. Bobeuf, F., Labonté, M., Dionne, I. J., & Khalil, A. (2011). Combined effect of antioxidant supplementation and resistance training on oxidative stress markers, muscle and body composition in an elderly population. The Journal of Nutrition, Health & Aging, 15(10), 883–889. https://doi.org/10.1007/s12603-011-0097-2

15. Bock, B. C., Dunsiger, S. I., Ciccolo, J. T., Serber, E. R., Wu, W. C., Tilkemeier, P., Walaska, K. A., & Marcus, B. H. (2019). Exercise videogames, physical activity, and health: Wii Heart Fitness, a randomized clinical trial. American Journal of Preventive Medicine, 56(4), 501–511. https://doi.org/10.1016/j.amepre.2018.11.026

16. Bonfante, I. L. P., Chacon-Mikahil, M. P. T., Brunelli, D. T., Gáspari, A. F., Duft, R. G., Lopes, W. A., Bonganha, V., Libardi, C. A., & Cavaglieri, C. R. (2017). Combined training, FNDC5/irisin levels and metabolic markers in obese men: A randomised controlled trial. European Journal of Sport Science, 17(5), 629–637. https://doi.org/10.1080/17461391.2017.1296025

17. Bonfante, I. L. P., Monfort-Pires, M., Duft, R. G., da Silva Mateus, K. C., de Lima Júnior, J. C., dos Santos Trombeta, J. C., Finardi, E. A. R., Brunelli, D. T., Morari, J., de Lima, J. A. B., Bellotto, M. L., de Araújo, T. M. F., Ramos, C. D., Chacon-Mikahil, M. P. T., Velloso, L. A., & Cavaglieri, C. R. (2022). Combined training increases thermogenic fat activity in patients with overweight and type 2 diabetes. International Journal of Obesity, 46(6), 1145–1154. https://doi.org/10.1038/s41366-022-01086-3

18. Boyden, T. W., Pamenter, R. W., Going, S. B., Lohman, T. G., Hall, M. C., Houtkooper, L. B., Bunt, J. C., Ritenbaugh, C., & Aickin, M. (1993). Resistance exercise training is associated with decreases in serum low-density lipoprotein cholesterol levels in premenopausal women. Archives of Internal Medicine, 153(1), 97–100. https://doi.org/10.1001/archinte.1993.00410010119011

19. Busby, J., Notelovitz, M., Putney, K., & Grow, T. (1985). Exercise, high-density lipoprotein-cholesterol, and cardiorespiratory function in climacteric women. Southern Medical Journal, 78(7), 769–773. https://doi.org/10.1097/00007611-198507000-00003

20. Cho, J. K., Lee, S. H., Lee, J. Y., & Kang, H. S. (2011). Randomized controlled trial of training intensity in adiposity. International Journal of Sports Medicine, 32(6), 468–475. https://doi.org/10.1055/s-0031-1271789

21. Colado, J. C., Triplett, N. T., Tella, V., Saucedo, P., & Abellán, J. (2009). Effects of aquatic resistance training on health and fitness in postmenopausal women. European Journal of Applied Physiology, 106(1), 113–122. https://doi.org/10.1007/s00421-009-0996-7

22. Dalleck, L. C., Allen, B. A., Hanson, B. A., Borresen, E. C., Erickson, M. E., & De Lap, S. L. (2009). Dose-response relationship between moderate-intensity exercise duration and coronary heart disease risk factors in postmenopausal women. Journal of Women's Health, 18(1), 105–113. https://doi.org/10.1089/jwh.2008.0790

23. Sunami, Y., Motoyama, M., Kinoshita, F., Mizooka, Y., Sueta, K., Matsunaga, A., Sasaki, J., Tanaka, H., & Shindo, M. (1999). Effects of low-intensity aerobic training on the high-density lipoprotein cholesterol concentration in healthy elderly subjects. Metabolism, 48(8), 984–988. https://doi.org/10.1016/S0026-0495(99)90194-4

24. Eguchi, Y., Ohta, M., Inoue, T., Honda, T., Morita, Y., Konno, Y., & Yamato, H. (2012). Effects of transitory stimulation interval exercise on physical function: A randomized controlled pilot study among Japanese subjects. Journal of UOEH, 34(4), 297–308. https://doi.org/10.7888/juoeh.34.297

25. Elliott, K. J., Sale, C., & Cable, N. T. (2002). Effects of resistance training and detraining on muscle strength and blood lipid profiles in postmenopausal women. British Journal of Sports Medicine, 36(5), 340–344. https://doi.org/10.1136/bjsm.36.5.340

26. Taniguchi, H., Tanisawa, K., Sun, X., Kubo, T., & Higuchi, M. (2016). Endurance exercise reduces hepatic fat content and serum fibroblast growth factor 21 levels in elderly men. The Journal of Clinical Endocrinology & Metabolism, 101(1), 191–198. https://doi.org/10.1210/jc.2015-3308

27. Fahlman, M. M., Boardley, D., Lambert, C. P., & Flynn, M. G. (2002). Effects of endurance training and resistance training on plasma lipoprotein profiles in elderly women. The Journals of Gerontology: Series A, 57(2), B54–B60. https://doi.org/10.1093/gerona/57.2.B54

28. Fenkci, S., Sarsan, A., Rota, S., & Ardic, F. (2006). Effects of resistance or aerobic exercises on metabolic parameters in obese women who are not on a diet. Advances in Therapy, 23(3), 404–413. https://doi.org/10.1007/BF02850161

29. Ha, M. S., Kim, J. H., Kim, Y. S., & Kim, D. Y. (2018). Effects of aquarobic exercise and burdock intake on serum blood lipids and vascular elasticity in Korean elderly women. Experimental Gerontology, 101, 63–68. https://doi.org/10.1016/j.exger.2017.11.005

30. Hagan, R. D., Upton, S. J., Wong, L., & Whittam, J. (1986). The effects of aerobic conditioning and/or caloric restriction in overweight men and women. Medicine & Science in Sports & Exercise, 18(1), 87–94. https://doi.org/10.1249/00005768-198602000-00015

31. Hallsworth, K., Thoma, C., Hollingsworth, K. G., Cassidy, S., Anstee, Q. M., Day, C. P., & Trenell, M. I. (2015). Modified high-intensity interval training reduces liver fat and improves cardiac function in non-alcoholic fatty liver disease: A randomized controlled trial. Clinical Science, 129(12), 1097–1105. https://doi.org/10.1042/CS20150308

32. Hersey, W. C., III, Graves, J. E., Pollock, M. L., Gingerich, R., Shireman, R. B., Heath, G. W., Spierto, F., McCole, S. D., & Hagberg, J. M. (1994). Endurance exercise training improves body composition and plasma insulin responses in 70- to 79-year-old men and women. Metabolism, 43(7), 847–854. https://doi.org/10.1016/0026-0495(94)90265-8

33. Hornstrup, T., Løwenstein, F. T., Larsen, M. A., Helge, E. W., Póvoas, S., Helge, J. W., Nielsen, J. J., Fristrup, B., Andersen, J. L., Gliemann, L., Nybo, L., & Krustrup, P. (2019). Cardiovascular, muscular, and skeletal adaptations to recreational team handball training: A randomized controlled trial with young adult untrained men. European Journal of Applied Physiology, 119(2), 561–573. https://doi.org/10.1007/s00421-018-4034-5

34. Houghton, D., Thoma, C., Hallsworth, K., Cassidy, S., Hardy, T., Burt, A. D., Tiniakos, D., Hollingsworth, K. G., Taylor, R., Day, C. P., McPherson, S., Anstee, Q. M., & Trenell, M. I. (2017). Exercise reduces liver lipids and visceral adiposity in patients with nonalcoholic steatohepatitis in a randomized controlled trial. Clinical Gastroenterology and Hepatology, 15(1), 96–102.e3. https://doi.org/10.1016/j.cgh.2016.07.031

35. Hsieh, P. L., Tseng, C. H., Tseng, Y. J., & Yang, W. S. (2018). Resistance training improves muscle function and cardiometabolic risks but not quality of life in older people with type 2 diabetes mellitus: A randomized controlled trial. Journal of Geriatric Physical Therapy, 41(2), 65–76. https://doi.org/10.1519/JPT.0000000000000107

36. Huttunen, J. K., Länsimies, E., Voutilainen, E., Ehnholm, C., Hietanen, E., Penttilä, I., Siitonen, O., & Rauramaa, R. (1979). Effect of moderate physical exercise on serum lipoproteins: A controlled clinical trial with special reference to serum high-density lipoproteins. Circulation, 60(6), 1220–1229. https://doi.org/10.1161/01.CIR.60.6.1220

37. Vicente-Campos, D., Mora, J., Castro-Piñero, J., González-Montesinos, J. L., Conde-Caveda, J., & Chicharro, J. L. (2012). Impact of a physical activity program on cerebral vasoreactivity in sedentary elderly people. The Journal of Sports Medicine and Physical Fitness, 52(5), 537–544.

38. Jennings, F., Oliveira, H. A., de Souza, M. C., Cruz, V. D. G., & Natour, J. (2015). Effects of aerobic training in patients with ankylosing spondylitis. The Journal of Rheumatology, 42(12), 2347–2353. https://doi.org/10.3899/jrheum.150518

39. Duan, Y., & Lu, G. (2024). A randomized controlled trial to determine the impact of resistance training versus aerobic training on the management of FGF-21 and related physiological variables in obese men with type 2 diabetes mellitus. Journal of Sports Science and Medicine, 23, 495–503. https://doi.org/10.52082/jssm.2024.495

40. Juneau, M., Rogers, F., de Santos, V., Yee, M., Evans, A., Bohn, A., Haskell, W. L., Taylor, C. B., & DeBusk, R. F. (1987). Effectiveness of self-monitored, home-based, moderate-intensity exercise training in middle-aged men and women. The American Journal of Cardiology, 60(1), 66–70. https://doi.org/10.1016/0002-9149(87)90986-6

41. Kantyka, J., Herman, D., Roczniok, R., & Kuba, L. (2015). Effects of aqua aerobics on body composition, body mass, lipid profile, and blood count in middle-aged sedentary women. Human Movement, 16(1), 9–14. https://doi.org/10.1515/humo-2015-0020

42. Kraus, W. E., Houmard, J. A., Duscha, B. D., Knetzger, K. J., Wharton, M. B., McCartney, J. S., Bales, C. W., Henes, S., Samsa, G. P., Otvos, J. D., Kulkarni, K. R., & Slentz, C. A. (2002). Effects of the amount and intensity of exercise on plasma lipoproteins. The New England Journal of Medicine, 347(19), 1483–1492. https://doi.org/10.1056/NEJMoa020194

43. LeMura, L. M., von Duvillard, S. P., Andreacci, J., Klebez, J. M., Chelland, S. A., & Russo, J. (2000). Lipid and lipoprotein profiles, cardiovascular fitness, body composition, and diet during and after resistance, aerobic and combination training in young women. European Journal of Applied Physiology, 82(5–6), 451–458. https://doi.org/10.1007/s004210000234

44. Liao, H. C., Zhong, S. G., Li, P., Chen, W. B., Cheng, C., Wang, Y. G., Wu, P. S., & Xiao, C. (2015). Effects and mechanism of moderate aerobic exercise on impaired fasting glucose improvement. Lipids in Health and Disease, 14, 157. https://doi.org/10.1186/s12944-015-0117-z

45. Libardi, C. A., de Souza, G. V., Cavaglieri, C. R., Madruga, V. A., & Chacon-Mikahil, M. P. T. (2012). Effect of resistance, endurance, and concurrent training on TNF-α, IL-6, and CRP. Medicine & Science in Sports & Exercise, 44(1), 50–56. https://doi.org/10.1249/MSS.0b013e318229d2e9

46. Manning, J. M., Dooly-Manning, C. R., White, K., Kampa, I., Silas, S., Kesselhaut, M., & Ruoff, M. (1991). Effects of a resistive training program on lipoprotein-lipid levels in obese women. Medicine & Science in Sports & Exercise, 23(11), 1222–1226. https://doi.org/10.1249/00005768-199111000-00003

47. Mendham, A. E., Duffield, R., Marino, F., & Coutts, A. J. (2015). A 12-week sports-based exercise programme for inactive Indigenous Australian men improved clinical risk factors associated with type 2 diabetes mellitus. Journal of Science and Medicine in Sport, 18(4), 438–443. https://doi.org/10.1016/j.jsams.2014.06.013

48. Morey, M. C., Pieper, C. F., Edelman, D. E., Yancy, W. S., Jr., Green, J. B., Lum, H., Peterson, M. J., Sloane, R., Cowper, P. A., Bosworth, H. B., Huffman, K. M., Cavanaugh, J. T., Hall, K. S., Pearson, M. P., & Taylor, G. A. (2012). Enhanced fitness: A randomized controlled trial of the effects of home-based physical activity counseling on glycemic control in older adults with prediabetes mellitus. Journal of the American Geriatrics Society, 60(9), 1655–1662. https://doi.org/10.1111/j.1532-5415.2012.04119.x

49. Motoyama, M., Sunami, Y., Kinoshita, F., Irie, T., Sasaki, J., Arakawa, K., Kiyonaga, A., Tanaka, H., & Shindo, M. (1995). The effects of long-term low-intensity aerobic training and detraining on serum lipid and lipoprotein concentrations in elderly men and women. European Journal of Applied Physiology and Occupational Physiology, 70(2), 126–131. https://doi.org/10.1007/BF00361539

50. Murphy, M. H., Murtagh, E. M., Boreham, C. A., Hare, L. G., & Nevill, A. M. (2006). The effect of a worksite-based walking programme on cardiovascular risk in previously sedentary civil servants. BMC Public Health, 6, 136. https://doi.org/10.1186/1471-2458-6-136

51. Murtagh, E. M., Boreham, C. A. G., Nevill, A., Hare, L. G., & Murphy, M. H. (2005). The effects of 60 minutes of brisk walking per week, accumulated in two different patterns, on cardiovascular risk. Preventive Medicine, 41(1), 92–97. https://doi.org/10.1016/j.ypmed.2004.10.008

52. Niederseer, D., Ledl-Kurkowski, E., Kvita, K., Patsch, W., Dela, F., Mueller, E., & Niebauer, J. (2011). Salzburg Skiing for the Elderly Study: Changes in cardiovascular risk factors through skiing in the elderly. Scandinavian Journal of Medicine & Science in Sports, 21(Suppl. 1), 47–55. https://doi.org/10.1111/j.1600-0838.2011.01341.x

53. Nieman, D. C., Brock, D. W., Butterworth, D., Utter, A. C., & Nieman, C. C. (2002). Reducing diet and/or exercise training decreases the lipid and lipoprotein risk factors of moderately obese women. Journal of the American College of Nutrition, 21(4), 344–350. https://doi.org/10.1080/07315724.2002.10719233

54. Nualnim, N., Parkhurst, K., Dhindsa, M., Tarumi, T., Vavrek, J., & Tanaka, H. (2012). Effects of swimming training on blood pressure and vascular function in adults older than 50 years. The American Journal of Cardiology, 109(7), 1005–1010. https://doi.org/10.1016/j.amjcard.2011.11.029

55. Park, S. K., Park, J. H., Kwon, Y. C., Kim, H. S., Yoon, M. S., & Park, H. T. (2003). The effect of combined aerobic and resistance exercise training on abdominal fat in obese middle-aged women. Journal of Physiological Anthropology and Applied Human Science, 22(3), 129–135. https://doi.org/10.2114/jpa.22.129

56. Park, S. Y., & Lee, I. H. (2015). Effects of training and detraining on physical function, control of diabetes and anthropometrics in type 2 diabetes: A randomized controlled trial. Physiotherapy Theory and Practice, 31(2), 83–88. https://doi.org/10.3109/09593985.2014.958265

57. Patterson, S., Pattison, J., Legg, H., Gibson, A. M., & Brown, N. (2017). The impact of badminton on health markers in untrained females. Journal of Sports Sciences, 35(11), 1098–1106. https://doi.org/10.1080/02640414.2016.1210819

58. Prabhakaran, B., Dowling, E. A., Branch, J. D., Swain, D. P., & Leutholtz, B. C. (1999). Effect of 14 weeks of resistance training on lipid profile and body fat percentage in premenopausal women. British Journal of Sports Medicine, 33(3), 190–195. https://doi.org/10.1136/bjsm.33.3.190

59. Raz, I., Rosenblit, H., & Kark, J. D. (1988). Effect of moderate exercise on serum lipids in young men with low high-density lipoprotein cholesterol. Arteriosclerosis, 8(3), 245–251. https://doi.org/10.1161/01.ATV.8.3.245

60. Rowland, S. A., Berg, K. E., Kupzyk, K. A., Pullen, C. H., Cohen, M. Z., Schulz, P. S., & Yates, B. C. (2018). Feasibility and effect of a peer modeling workplace physical activity intervention for women. Workplace Health & Safety, 66(9), 428–436. https://doi.org/10.1177/2165079917753690

61. Herawati, I., Mat Ludin, A. F., Ishak, I., Mutalazimah, & Farah, N. M. F. (2025). Impact of combined high-intensity bodyweight interval training and breathing exercise on cardiometabolic health in normal-weight middle-aged adults with hypertension. BMC Public Health, 25, 962. https://doi.org/10.1186/s12889-025-22139-y

62. Schuit, A. J., Schouten, E. G., Miles, T. P., Evans, W. J., Saris, W. H. M., & Kok, F. J. (1998). The effect of six months training on weight, body fatness and serum lipids in apparently healthy elderly Dutch men and women. International Journal of Obesity, 22(9), 847–853. https://doi.org/10.1038/sj.ijo.0800671

63. Smith-Ryan, A. E., Melvin, M. N., & Wingfield, H. L. (2015). High-intensity interval training: Modulating interval duration in overweight/obese men. The Physician and Sportsmedicine, 43(2), 107–113. https://doi.org/10.1080/00913847.2015.1037231

64. Sousa, N., Mendes, R., Abrantes, C., Sampaio, J., & Oliveira, J. (2014). A randomized study on lipids response to different exercise programs in overweight older men. International Journal of Sports Medicine, 35(13), 1106–1111. https://doi.org/10.1055/s-0034-1374639

65. Stensvold, D., Tjønna, A. E., Skaug, E. A., Aspenes, S., Stølen, T., Wisløff, U., & Slørdahl, S. A. (2010). Strength training versus aerobic interval training to modify risk factors of metabolic syndrome. Journal of Applied Physiology, 108(4), 804–810. https://doi.org/10.1152/japplphysiol.00996.2009

66. Suter, E., Marti, B., & Gutzwiller, F. (1994). Jogging or walking—Comparison of health effects. Annals of Epidemiology, 4(5), 375–381. https://doi.org/10.1016/1047-2797(94)90072-8

67. Takeshima, N., Rogers, M. E., Islam, M. M., Yamauchi, T., Watanabe, E., & Okada, A. (2004). Effect of concurrent aerobic and resistance circuit exercise training on fitness in older adults. European Journal of Applied Physiology, 93(1–2), 173–182. https://doi.org/10.1007/s00421-004-1193-3

68. Ho, S. S., Dhaliwal, S. S., Hills, A. P., & Pal, S. (2012). The effect of 12 weeks of aerobic, resistance or combination exercise training on cardiovascular risk factors in the overweight and obese in a randomized trial. BMC Public Health, 12, 704. https://doi.org/10.1186/1471-2458-12-704

69. Thompson, D., Markovitch, D., Betts, J. A., Mazzatti, D., Turner, J., & Tyrrell, R. M. (2010). Time course of changes in inflammatory markers during a 6-month exercise intervention in sedentary middle-aged men: A randomized-controlled trial. Journal of Applied Physiology, 108(4), 769–779. https://doi.org/10.1152/japplphysiol.00822.2009

70. Tokudome, M., Nagasaki, M., Shimaoka, K., & Sato, Y. (2004). Effects of home-based combined resistance training and walking on metabolic profiles in elderly Japanese. Geriatrics & Gerontology International, 4(3), 157–162. https://doi.org/10.1111/j.1447-0594.2004.00241.x

71. Vinetti, G., Mozzini, C., Desenzani, P., Boni, E., Bulla, L., Lorenzetti, I., Romano, C., Pasini, A., Cominacini, L., & Assanelli, D. (2015). Supervised exercise training reduces oxidative stress and cardiometabolic risk in adults with type 2 diabetes: A randomized controlled trial. Scientific Reports, 5, 9238. https://doi.org/10.1038/srep09238

72. Wen, H. J., Huang, T. H., Li, T. L., Chong, P. N., & Ang, B. S. (2017). Effects of short-term step aerobics exercise on bone metabolism and functional fitness in postmenopausal women with low bone mass. Osteoporosis International, 28(2), 539–547. https://doi.org/10.1007/s00198-016-3759-4

73. Kim, W. C., Choi, S. L., Kim, S. W., & Park, H. R. (2016). The effects of aquarobics on blood pressure, heart rate, and lipid profile in older women with hypertension. Indian Journal of Science and Technology, 9(46), 1–7. https://doi.org/10.17485/ijst/2016/v9i46/107177

74. Woolf-May, K., Kearney, E. M., Jones, D. W., Davison, R. C. R., Coleman, D., & Bird, S. R. (1998). The effect of two different 18-week walking programmes on aerobic fitness, selected blood lipids and factor XIIa. Journal of Sports Sciences, 16(8), 701–710. https://doi.org/10.1080/026404198366335

75. Wooten, J. S., Phillips, M. D., Mitchell, J. B., Patrizi, R., Pleasant, R. N., Hein, R. M., Menzies, R. D., & Barbee, J. J. (2011). Resistance exercise and lipoproteins in postmenopausal women. International Journal of Sports Medicine, 32(1), 7–13. https://doi.org/10.1055/s-0030-1268008

76. Wu, Y. T., Hwang, C. L., Chen, C. N., & Chuang, L. M. (2011). Home-based exercise for middle-aged Chinese at diabetic risk: A randomized controlled trial. Preventive Medicine, 52(5), 337–343. https://doi.org/10.1016/j.ypmed.2011.02.018
